# National and State Societal Costs of Schizophrenia in the US in 2024

**DOI:** 10.1001/jamapsychiatry.2025.4383

**Published:** 2026-01-28

**Authors:** Holly B. Krasa, James R. Baumgardner, Iris P. Brewer, Jacquelyn W. Chou, Thomas Flottemesch, Jessica T. Markowitz, Cory Williams, Arundati Nagendra

**Affiliations:** 1 Schizophrenia & Psychosis Action Alliance, Alexandria, Virginia; 2 Blue Persimmon Group, Washington, DC; 3 Precision AQ, Bethesda, Maryland

## Abstract

**Importance:**

Schizophrenia imposes a substantial burden on individuals and society. Population-specific cost estimates are essential to inform evidence-based policy, allocate resources, and support recovery-focused care that improves outcomes.

**Objective:**

To estimate national and state-level burden of schizophrenia to inform population-specific care and services in the US in 2024.

**Design, Setting, and Participants:**

This was an observational prevalence-based cost-of-illness model estimating total excess direct medical, direct nonmedical, and indirect costs of schizophrenia by combining inputs from a targeted literature review and an analysis of Medical Expenditure Panel Survey data, adjusted to 2024 US dollars. The setting included independent households, supportive housing, long-term care and skilled nursing facilities, unhoused settings, and prisons and jails. Disease-related costs were estimated for adults living with schizophrenia spectrum disorders.

**Exposures:**

Health care, supportive housing, homelessness, social security disability benefits, justice system, employment, productivity, quality of life, mortality, and caregiver impact across settings of care.

**Main Outcomes and Measures:**

Prevalence-based national and state cost of schizophrenia by category or sector.

**Results:**

The societal cost of schizophrenia in 2024 was estimated at $366.8 billion in the US for 3 070 739 adults (1.17%) across all settings (68.4% independent households, 18.6% supportive housing, 5.0% long-term care or skilled nursing facility, 4.7% incarcerated, 3.3% unhoused). Direct costs ($75.0 billion) were attributable to health care ($36.7 billion), supportive housing and homelessness ($35.2 billion), justice system interactions ($11.9 billion), and social security disability benefits ($5.1 billion). Indirect costs ($291.8 billion) resulted from lost wages ($55.4 billion) and reduced quality of life ($41.4 billion) and life expectancy ($47.5 billion). Indirect costs for caregivers of individuals with schizophrenia included unpaid wages for time providing care ($104.6 billion) and impact on caregiver health, productivity, and out-of-pocket costs ($60.5 billion). Per-person costs of schizophrenia were estimated at $119 436 nationally in 2024. State-level per-person costs ranged from $110 975 in Utah to $126 225 in Alaska.

**Conclusions and Relevance:**

In 2024, the national and state-level costs of schizophrenia in the US estimated from a societal perspective suggest a substantial burden of disease on individuals, families, and society. These findings provide an important framework to guide prevention, care, and management strategies to reduce costs and improve public health outcomes.

## Key Points

**Question:**

What was the economic burden of schizophrenia in 2024 in the US?

**Findings:**

In this economic evaluation representing an estimated 3 070 739 adults living with schizophrenia spectrum disorders in the US, the 2024 societal burden of schizophrenia was estimated at $366.8 billion, with per-person costs of $119 436. Health care made up less than half of direct costs and just 9% of the total; indirect costs including lost productivity, premature mortality, and unpaid caregiving accounted for the largest share.

**Meaning:**

These findings suggest a substantial, multisector schizophrenia-related economic burden with state-level variation, highlighting the need for coordinated care and cross-sector responses.

## Teaser

This economic evaluation investigates the societal burden of schizophrenia in the US in 2024, including health care, housing, employment, justice system, and caregiver impacts.

## Introduction

Schizophrenia spectrum disorders (schizophrenia) are serious neuropsychiatric diseases, characterized by hallucinations, delusions, disordered thinking, and cognitive impairment.^1,2,3^ Symptoms emerge in adolescence or early adulthood and have lifelong impacts on individuals, families, and society.^1,2,3^ Schizophrenia affects approximately 1% of the population and is a leading cause of disability globally.^4^ Suboptimal care and treatment are associated with functional challenges and disability, which lead to reduced life expectancy, high unemployment and underemployment, homelessness, frequent encounters with the justice system, heavy use of supportive services (eg, housing and transportation), and increased caregiver burden.^5,6,7,8,9,10,11,12,13,14^

The resulting direct and indirect economic burden of schizophrenia is high, with estimates of annual costs in the US more than doubling between 2013 and 2019 from $155.7 billion to $342.3 billion.^15,16^ These prior estimates underscore the need for societal action but fail to account for important population and regional heterogeneity. For example, estimates using retrospective, claims-based analyses often exclude the uninsured or do not fully capture health care–related utilization (HCRU) for the partially insured.^16,17,18^ Nationally representative surveys exclude institutionalized and unhoused individuals.^19^ Prospective, longitudinal investigations are limited in sample size, geography, and generalizability.^20^ In addition, prior estimates relied on outdated or incomplete cost and prevalence data, with limited adjustments for key population characteristics (eg, age and residential setting), limiting utility for local policymakers.^15^

This study builds on prior reports of the societal burden of schizophrenia by incorporating updated disease prevalence and cost data, quantifying disease-related costs not captured in earlier models, and generating state and per-person estimates. These geographically targeted analyses complement national totals and provide policymakers and health systems with actionable, population-specific information not available in previous studies.

## Methods

### Overview

This economic evaluation was not subject to institutional review under 45 Code of Federal Regulations part 46 as human participants and identifiable private information were not involved. This study followed the Consolidated Health Economic Evaluation Reporting Standards (CHEERS) reporting guidelines.

**Figure 1.**
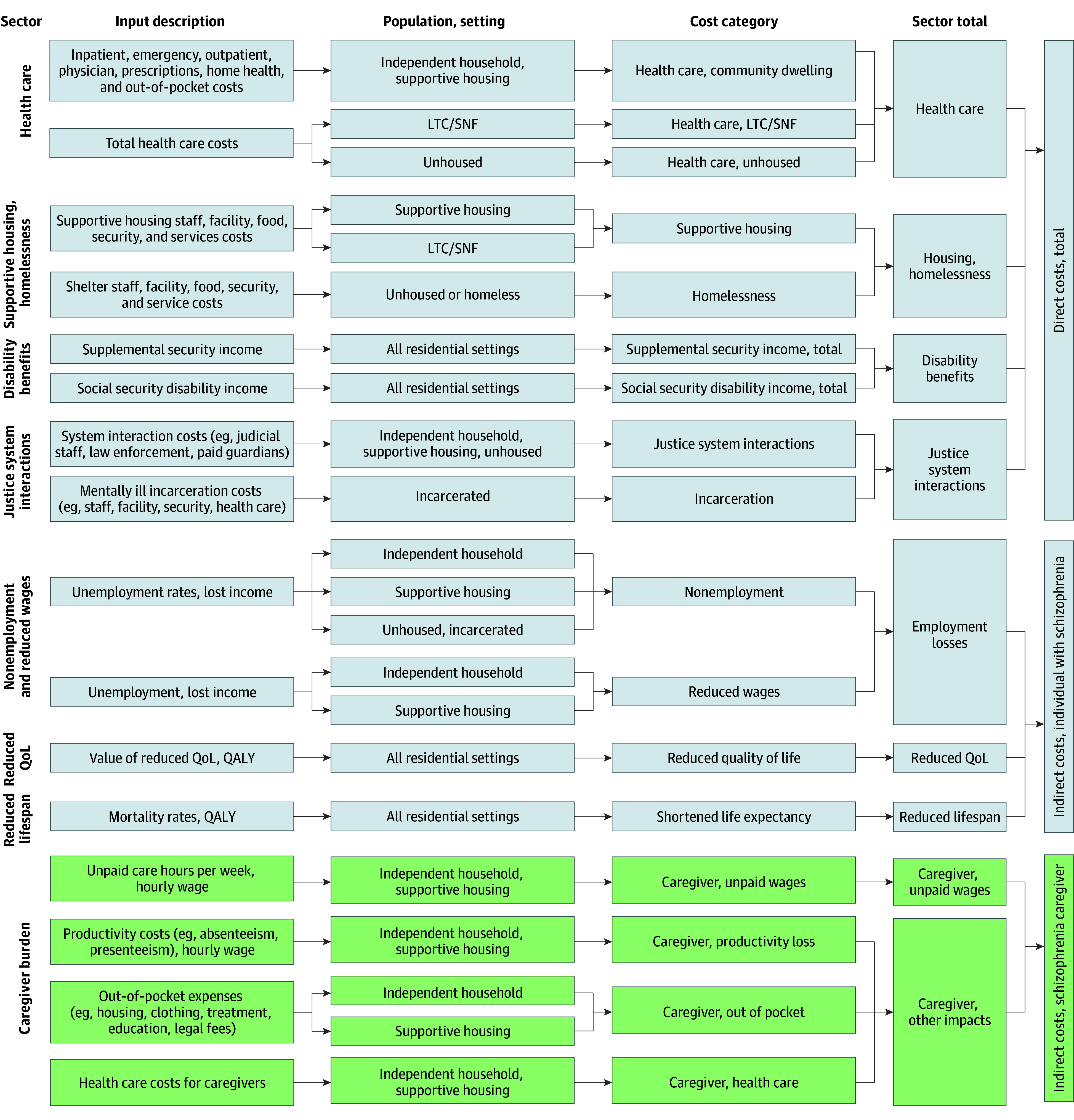
Framework for Estimating the Societal Costs of Schizophrenia in the US Left to right, the figure shows how cost inputs are adjusted to 2024 dollars, converted to excess costs, applied to the relevant prevalent population with schizophrenia by residential setting (independent household, supportive housing, long-term care [LTC] or skilled nursing facility [SNF], unhoused, incarcerated, unhoused), grouped into cost categories and domains, and summed to national and state direct, indirect, and total costs for 2024. Model sources by input and calculation descriptions are provided in eTable 10 to 18 in Supplement 1. QALY indicates quality-adjusted life-year; QoL, quality of life.

A multidisciplinary approach was leveraged to quantify direct medical, direct nonmedical, and indirect societal costs of schizophrenia nationally and by state and the District of Columbia. Targeted literature reviews identified overall and location-specific prevalence and disease-attributable outcomes and costs. An empirical analysis of pooled data from the Medical Expenditure Panel Survey (MEPS) informed excess direct medical and indirect employment costs for community dwelling individuals.^21^ Excess costs (ie, disease related or incremental) were defined as the additional costs incurred by adults with schizophrenia compared with those without the condition. A prevalence-based, cross-sectional model from a societal perspective then combined these inputs to estimate the total economic burden of schizophrenia among US adults (18 years and older) across community, institutional, and unhoused residential settings for the calendar year 2024 (Figure 1). Cost inputs were inflated to 2024 US dollars ($2024) using an appropriate index: the Personal Consumption Expenditures (PCE) Price Index, the Personal Health Care Component of the PCE Price Index (PHCE), the Consumer Price Index–Urban Wage Earners and Clerical Workers, or the Business Sector: Hourly Compensation for All Workers (HCOMP-BS) (eAppendix 1 and the eFigure in Supplement 1).^22,23,24,25^ Outcomes were estimated by domain: health care, supportive housing and homelessness, social security disability benefits, justice system interactions, employment, morbidity and mortality, and caregiver burden (eAppendix 2 in Supplement 1).

### Data Sources

#### Targeted Literature Reviews

Literature searches were limited to US-based, English-language studies published after 2011 that identified disease-attributable HCRU, housing and homelessness, social security disability benefits, justice system interactions, employment and productivity, morbidity and mortality, and caregiver burden within the peer-reviewed and gray literature (eAppendix 1 and eTable 1 in Supplement 1). Preference was given to studies published since 2020 and with national or multisite designs. Where available, state-specific data was identified. Gray literature included governmental (eg, datasets, reports, publications, and surveys) and other publicly available sources (eg, white papers, reports, datasets).

#### Community Health Care and Employment Costs

An analysis of a pooled (2006-2015) sample from MEPS with costs adjusted to $2015 by the PHCE used a quasi-experimental, matched, case-control design. Years after 2015 were excluded because MEPS discontinued schizophrenia-specific identifiers. Cases (ie, diagnosed schizophrenia) were identified by the clinical classification system to estimate excess health care costs and productivity losses attributable to schizophrenia among those in a community setting using weighted regressions, adjusting for demographics, geography, and insurance coverage (eTable 2-9 in Supplement 1).^21,26^ Final HCRU estimates were adjusted to $2024 by the PHCE and productivity losses by the HCOMP-BS.^23,25^ These estimates informed direct medical costs and indirect employment costs among those living in a community setting (ie, independent household or supportive housing).

### Main Outcomes and Measures

#### Prevalence by Setting

An estimated population-wide prevalence of 1.17% combined published, age-based estimates for adults aged 18 to 64 years with a downward adjustment for early mortality risk and early-onset dementia among those 65 years and older.^27,28^ This population-wide estimate was distributed across places of residence based on the total number of adults in each setting and the estimated prevalence of schizophrenia among that setting’s subpopulation.^5,28,29,30,31,32,33,34,35^ Settings included independent households, supportive housing (eg, supervised apartment programs, boarding homes, halfway houses, treatment programs, or psychiatric diversion facilities), long-term care and skilled nursing facilities (long-term care [LTC]/skilled nursing facility [SNF]), unhoused, and prisons or jails. Any remaining adults with schizophrenia were assumed to be living in independent households (eTable 10 in Supplement 1). This study did not estimate costs of disease by demographic categories, including race and ethnicity, as many of the source references did not report demographic information.

#### Direct Costs

Direct medical costs included schizophrenia-related HCRU, professional services, and remediation costs attributable to schizophrenia (eTable 11 in Supplement 1). Health care costs are based on the MEPS analysis for community dwelling individuals in independent households and supportive housing. Literature-based estimates quantified medical costs among those in LTC/SNF and the homeless.^15,36^

Possible incremental direct nonmedical costs incurred by all with schizophrenia were estimated for disease-attributable Supplemental Security Income and/or Social Security Disability Income (SSDI) and justice system interactions (eg, services provided by law enforcement, judicial staff, institutions, and paid guardians).^37,38,39^ Costs of housing (eg, staff, facility costs, security, and food) were specific to individuals in LTC/SNF and supportive housing settings, shelter stays and remediation costs specific to the homeless, and incarceration costs (eg, staff, facility costs, security, food, and health care) specific to the incarcerated (eTable 12-14 in Supplement 1).^37,40,41,42^

#### Indirect Costs

Indirect costs among those with schizophrenia such as unemployment and underemployment, reduced quality of life (morbidity), and shortened life expectancy (mortality) were included as lost opportunity costs (eTable 15-17 in Supplement 1).^10,43,44,45,46,47,48,49^ A base case discount rate of 3% was applied to future lost years of life.^50^ Indirect costs for unpaid caregivers included uncompensated labor (ie, mean US wage for caregiver time) and lost productivity (ie, caregiver absenteeism or presenteeism labor cost). Other caregiver impacts included the economic burden of added health care costs and out-of-pocket costs borne by caregivers for everyday expenses (eg, food, transportation, housing, and property damage) and significant life events (eg, homelessness, substance use treatment, or the need for legal, education, or employment support).^12,16,25,44,49^ Caregiver costs were adjusted by HCOMP-BS to $2024 and estimated for schizophrenia populations residing in the community with a caregiver (eTable 18 in Supplement 1).^12,16,25,44,49^

#### Cost Offsets

Cost-of-living offsets using the US individual poverty threshold were applied to applicable direct and indirect costs to acknowledge individuals with schizophrenia would incur baseline expenses regardless of diagnosis.^16,42^

#### State-Level Adjustments

Where state-specific parameter values were unavailable, national estimates were adjusted to state values using Center for Medicare and Medicaid Services Geographic Practice Cost Indices (GPCIs) or Bureau of Labor Statistics reported state average wage-adjusted income and earnings.^51,52^ For the medical cost estimates, an average of the Practice Expense and Physician Wage GPCIs was used to create a state index. The indices were developed by first calculating a population-weighted average across all Medicare administrative contractor areas within the state. Then, these weighted averages were normalized so the average across all states equaled 1. For productivity losses, a similarly normalized index based on each state’s average wage relative to the national average was developed.

### Statistical Analysis

#### Societal Cost Estimates

Model parameters were combined to produce an economic burden estimate by cost area for both national and state populations using an excess cost approach.^53^ Excess costs were estimated using 2 approaches: (1) per-person differentials comparing adults with and without schizophrenia and (2) application of general-population unit costs or rates to the additional number of adults with schizophrenia using a given service. Totals were calculated by multiplying these estimates by the relevant population in each setting to reflect the economic burden of schizophrenia in the US in 2024. As all results reflect excess costs, the term *costs* is used hereafter. A separate scenario analysis to estimate lifetime costs for an individual diagnosed with schizophrenia at age 18 years was conducted by estimating per-person per-year annual costs averaged over a lifetime (eTable 19 in Supplement 1).

#### Sensitivity Analysis

A univariate sensitivity analysis of national estimated burden was conducted to determine how much individual parameters influenced the total societal cost estimates produced by the model. All model parameters were varied by an arbitrary ±20% from the base case with parameters resulting in at least a one-billion-dollar change identified. Data were analyzed from April 2024 to May 2025 using Microsoft Excel, version 2024 (Microsoft Corporation).

## Results

### National Cost of Schizophrenia

**Figure 2.**
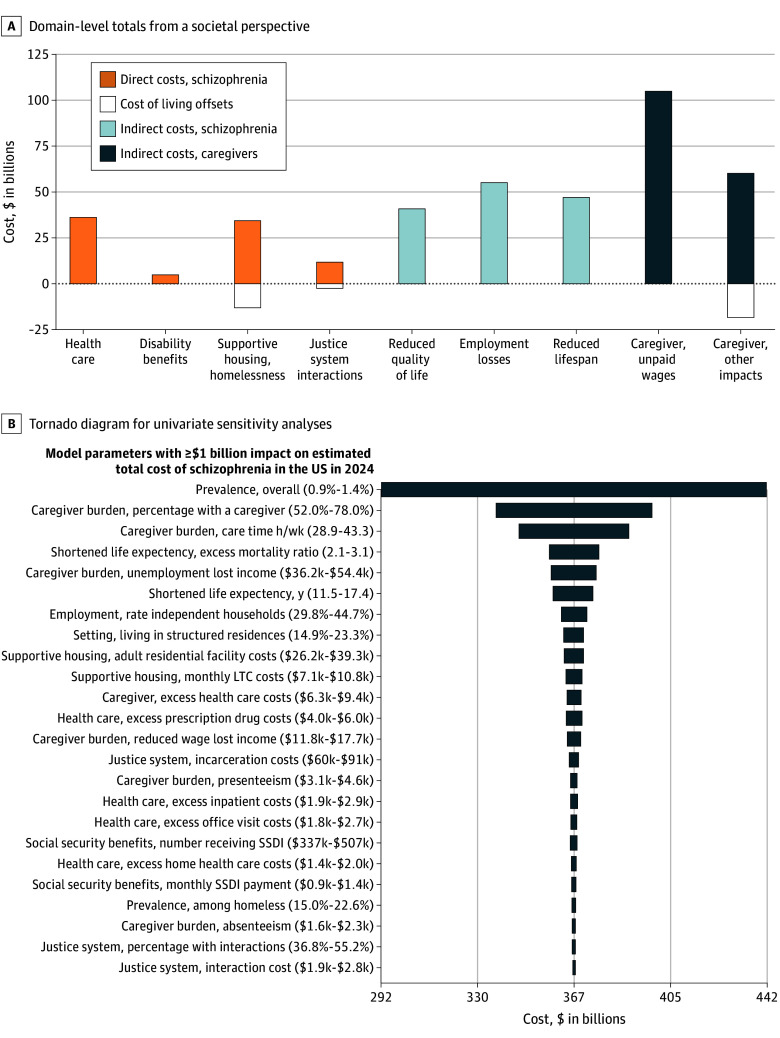
Societal Costs of Schizophrenia in the US in 2024 A, Domain-level totals from a societal perspective. Health care includes costs for individuals that are in independent households, supportive housing, unhoused, and in long-term care (LTC) settings. Supportive housing and homelessness include homeless shelter stays, supportive housing (residential and LTC facilities), and associated cost-of-living offsets. Justice system interactions include incarceration costs (eg, housing, living expenses, health care), justice system interactions, and cost-of-living offsets. Caregiver and other impacts include excess costs for caregiver health care, caregiver productivity loss, out-of-pocket costs, and living expense transfer cost offsets. B, Tornado diagram for univariate sensitivity analyses. All values represent annual average in 2024 and are specific to individuals with schizophrenia unless otherwise noted. The importance of each variable is presented from top to bottom. The maximum and minimum values for each variable, which were varied by ±20%, are presented in brackets. The tails of each bar indicate the maximum and minimum total societal cost of schizophrenia for each variable. The dashed line represents the total cost estimate from the reference case ($366.8 billion). Caregiver burden estimates were only applied to the proportion of individuals with schizophrenia living in independent households or supportive housing with a caregiver. SSDI indicates social security disability income.

In 2024, the total economic burden attributable to adults living with schizophrenia in the US was estimated at $366.8 billion for 3 070 739 adults based on an age-adjusted prevalence of 1.17% (Figure 2 and Table 1). Among the approximately 3.1 million adults living with schizophrenia, it was estimated that 68.4% reside in independent households, 18.6% in community-based structured residential facilities, 5.0% in LTC/SNF, 4.7% are incarcerated, and 3.3% are unhoused.

**Table 1.** Societal Cost of Schizophrenia in the US, 2024

Estimate^a^	US	Alaska	California	Illinois	Louisiana
Total population, prevalence, No. (%)	340 110 998 (100)	741 485 (100)	39 394 446 (100)	12 687 940 (100)	4 624 135 (100)
Adult population	263 249 065 (77.4)	569 070 (76.7)	31 357 812 (79.6)	10 189 748 (80.3)	3 647 949 (78.9)
Schizophrenia adult population	3 070 739 (1.2)	6638 (1.2)	365 782 (1.2)	118 861 (1.2)	42 552 (1.2)
Estimated prevalence of schizophrenia by living situation, No. (%)					
Community dwelling, independent household^b^	2 100 079 (68.4)	4515 (68.0)	250 989 (68.6)	81 631 (68.7)	28 859 (67.8)
Community dwelling, structured residence^a^^,^^c^	571 157 (18.6)	1235 (18.6)	68 035 (18.6)	22 108 (18.6)	7915 (18.6)
Long-term care/skilled nursing facility^a^	154 000 (5.0)	333 (5.0)	18 344 (5.0)	5961 (5.0)	2134 (5.0)
Incarcerated^d^	143 703 (4.7)	311 (4.7)	17 118 (4.7)	5562 (4.7)	1991 (4.7)
Unhoused or homeless^d^	101 799 (3.3)	245 (3.7)	11 296 (3.1)	3599 (3.0)	1653 (3.9)
Estimated prevalence of schizophrenia by outcome, No. (%)					
Unemployed, total schizophrenia	2 061 502 (67.1)	4276 (64.4)	241 677 (66.1)	77 121 (64.9)	28 736 (67.5)
Unemployed, excess schizophrenia	944 061 (30.7)	1860 (28.0)	108 569 (29.7)	33 867 (28.5)	13 251 (31.1)
Excess mortality	47 110 (1.5)	76 (1.1)	4477 (1.2)	1803 (1.5)	760 (1.8)
With a caregiver	1 736 304 (56.5)	3737 (56.3)	207 366 (56.7)	67 430 (56.7)	23 903 (56.2)
Excess societal costs of schizophrenia, $ thousand					
Total excess societal costs^e^	366 755 997	837 891	45 572 807	14 245 805	4 985 357
Direct excess societal costs^e^	74 990 291	216 322	10 104 049	216 322	10 104 049
Health care, total	36 693 641	102 322	4 910 268	1 416 626	480 449
Health care, community	34 299 681	95 304	4 599 703	1 327 778	446 697
Health care, long-term care/skilled nursing facility	1 341 921	3745	179 488	51 776	17 591
Health care, unhoused	1 052 040	3273	131 077	37 071	16 161
Supplemental security benefits, total	5 127 004	12 556	562 301	178 532	85 176
Supplemental security income	847 610	1832	100 966	32 809	11 746
Social security disability insurance	4 279 394	10 724	461 335	145 723	73 431
Supportive housing and homelessness, total	35 165 901	98 454	4 666 892	1 353 044	466 045
Structured residences	18 691 540	52 164	2 500 073	721 187	245 031
Long-term care/skilled nursing facilities	14 771 410	41 224	1 975 739	569 934	193 641
Homeless shelters	1 702 951	5066	191 079	61 923	27 373
Justice system, total	11 942 030	33 327	1 597 298	460 766	156 550
Justice system interactions	2 789 826	7786	373 151	107 641	36 572
Incarceration	9 152 204	25 542	1 224 146	353 125	119 978
Cost-of-living offsets, ($ thousand)^e^	(13 938 286)	(30 336)	(1 632 710)	(536 314)	(197 655)
Indirect excess societal costs,^e^	291 765 707	621 569	35 468 758	11 373 151	3 994 791
Nonemployment, total	42 761 333	93 626	6 017 547	1 637 158	509 945
Nonemployment, independent households	25 055 488	51 101	3 482 770	926 641	295 294
Nonemployment, supportive housing	13 170 745	30 822	1 903 018	533 679	155 324
Nonemployment, incarcerated or unhoused	4 535 100	11 703	631 759	176 838	59 327
Reduced wages, total	12 635 958	33 319	1 912 085	564 174	146 652
Reduced wages, independent households	11 564 237	30 479	1 750 402	516 507	134 118
Reduced wages, supportive housing	1 071 720	2840	161 682	47 667	12 534
Reduced quality of life value	41 423 711	89 903	4 949 867	1 603 691	572 557
Shortened life expectancy value	47 459 081	76 350	4 510 529	1 816 664	765 590
Caregiver burden, total	165 033 655	366 093	20 175 963	6 433 563	2 241 192
Unpaid wages	104 587 913	225 100	12 490 864	4 061 730	1 439 828
Productivity loss	9 988 133	23 888	1 459 679	413 974	116 826
Health care	13 581 849	37 738	1 821 372	525 768	176 881
Out-of-pocket expenses	36 875 760	79 366	4 404 047	1 432 091	507 657
Caregiver, transfer costs, ($ thousand)^e^	(17 548 031)	(37 723)	(2 097 233)	(682 098)	(241 145)

^a^ Percentage based on actual national model parameter estimate. Alaska, California, Illinois, and Louisiana were chosen to represent the range of cost of living across the US.

^b^ Includes adults living independently alone, with family, or with others.

^c^ Includes adults in supervised and partially supervised housing, supportive housing programs, or group homes.

^d^ Number is based on actual national and state parameter inputs.

^e^ Costs were adjusted to deduct cost-of-living expenses that an individual with schizophrenia would typically incur had they not been homeless, living in supportive housing or long-term care, housed in a prison or jail, or in need of unpaid caregiver support.

Total direct costs in 2024 associated with schizophrenia were estimated at $75.0 billion, representing 20.4% of the total societal burden of schizophrenia. Notably, health care spending accounted for less than half of these direct costs. Of the total, $34.3 billion was incurred by community-dwelling individuals (those in independent households or supportive housing), $1.3 billion by individuals in LTC/SNF, and $1.1 billion by unhoused individuals. The majority of direct costs included $35.2 billion for supportive housing and homelessness remediation, $5.1 billion in supplemental security disability benefits, and $11.9 billion related to justice system interactions, with $14.0 billion in cost-of-living offsets.

Consistent with previous estimates, the indirect burden of schizophrenia was more than 3 times that of direct costs, accounting for 79.6% of overall societal burden ($291.8 billion). Nonemployment and reduced wages for individuals with schizophrenia accounted for $55.4 billion in societal costs. Quality-of-life impacts were valued at $41.4 billion, with an additional $47.5 billion from shortened life expectancy. The economic burden of caregiver-related impacts accounted for $165.0 billion, including unpaid wages ($104.6 billion), out-of-pocket expenses ($36.9 billion), health care costs ($13.6 billion), and lost productivity ($10.0 billion). Cost offsets for living expenses and out-of-pocket spending by caregivers for their care recipient contributed to a $17.5 billion reduction in estimated total indirect and overall societal costs.

In 2024, schizophrenia-related cost per diagnosed adult in any setting in the US was estimated at $119 436 ($24 421 direct, $95 015 indirect) (Table 2). Among individuals living in independent households, health care was the largest contributor to direct costs ($12 840), with caregiver burden accounting for the majority of indirect costs ($84 942).

**Table 2.** Per-Person Economic Burden for Adults with Schizophrenia, 2024^a^

Per adult with schizophrenia, $	US	Alaska	California	Illinois	Louisiana
Excess total costs	119 436	126 225	124 590	119 853	117 158
Excess direct costs	24 421	32 588	27 623	24 168	23 279
Excess indirect costs	95 015	93 637	96 967	95 684	93 879
Excess direct costs by category and parameter, per affected adult with schizophrenia^b^					
Health care, community dwelling	12 840	16 577	14 418	12 799	12 147
Inpatient hospital	2404	3103	2699	2396	2274
Outpatient	925	1194	1038	922	875
Office visits	2230	2879	2504	2223	2110
Emergency department	181	233	203	180	171
Home health care	1681	2170	1888	1676	1590
Prescription drugs	5069	6545	5692	5053	4796
Out of pocket	350	452	393	349	331
Health care, long-term care/skilled nursing	8714	11 249	9784	8686	8243
Health care, unhoused	10 334	13 342	11 604	10 301	9776
Supplemental security income	3159	3159	3159	3159	3159
Social security disability insurance	10 150	10 555	9862	9777	10 726
Housing, supportive housing	32 726	42 249	36 747	32 621	30 959
Housing, long-term care/skilled nursing	95 918	123 830	107 704	95 611	90 739
Homelessness, shelters	16 729	20 652	16 916	17 207	16 559
Cost-of-living offsets, supportive housing and homelessness^c^	42 654	42 036	41 249	43 105	43 309
Justice system interactions	1975	2550	2218	1969	1868
Incarceration^d^	63 688	82 127	71 513	63 489	60 260
Cost-of-living offsets, incarceration^c^	12 697	12 682	12 697	12 698	12 699
Excess indirect costs by category and parameter, per affected adult with schizophrenia^b^					
Nonemployment	20 743	21 897	24 899	21 228	17 746
Reduced wages	14 775	16 422	18 079	15 768	12 553
Reduced quality of life value	13 490	13 544	13 532	13 492	13 455
Shortened life expectancy value	15 455	11 502	12 331	15 284	17 992
Caregiver, unpaid wages	60 236	60 236	60 236	60 236	60 236
Caregiver, productivity loss	5753	6392	7039	6139	4887
Caregiver, health care	7822	10 099	8783	7797	7400
Caregiver, out-of-pocket	21 238	21 238	21 238	21 238	21 238
Caregiver, transfer costs^c^	(10 107)	(10 094)	(10 114)	(10 116)	(10 088)

^a^ Alaska, California, Illinois, and Louisiana were chosen to represent the range of cost of living across the US.

^b^ Excess cost per affected adult with schizophrenia in setting or by category/parameter.

^c^ Totals adjusted to deduct cost-of-living expenses that an individual with schizophrenia would typically incur had they not been homeless, living in supportive housing or long-term care, housed in a prison or jail, or in need of unpaid caregiver support.

^d^ Includes all costs associated with incarceration including health care.

A scenario analysis to determine lifetime cost, excluding the costs associated with reduced life expectancy, estimated a cost per adult of $103 980 per year. Based on an adjusted life expectancy of 44.6 years for an individual diagnosed at age 18 years, the adult lifetime economic burden of a person with schizophrenia was $4.5 million or $2.5 million in present value discounted at 3%.

Sensitivity analyses identified 24 model parameters that had an impact of at least $1 billion on the estimated total cost of schizophrenia in the US in 2024 (Figure 2). The most influential parameters (ie, those with an impact of at least $10 billion on total societal costs in the sensitivity analysis) were disease prevalence (±$145.3 billion), the percentage of individuals with a caregiver (±$59.0 billion), average caregiver hours per week (±$41.8 billion), mortality rate (±$18.7 billion), caregiver nonemployment lost income (±$17.1 billion), and reduced life expectancy (±$15.2 billion). Additional cost parameters with at least a $5 billion impact on total societal costs were linked to supportive housing (ie, number in setting and costs of living in supportive housing), costs of prescription drugs, employment rates, and caregiver impacts (ie, costs for caregiver health care and productivity losses).

### State Costs of Schizophrenia

State-level estimates reflected differences in population size. After adjusting for state-specific data and cost indices, total costs of schizophrenia ranged in 2024 from $45.6 billion in California (n = 365 782 adults with schizophrenia) to $0.6 billion in Wyoming (n = 5331) (Table 3). Per-person costs varied from $110 975 in Utah to $126 225 in Alaska, largely due to cost-of-living differences and local variations in how costs are incurred.

**Table 3.** State-Level Economic Burden for Adults with Schizophrenia, 2024

Population	Estimated No.	Total cost			
		Per person, $	Societal, $ thousand	Direct, $ thousand	Indirect, $ thousand
US	3 070 739	119 436	366 755 997	74 990 291	291 765 707
Alabama	46 857	118 844	5 568 632	1 084 636	4 483 996
Alaska	6638	126 225	837 891	216 323	621 569
Arizona	66 745	118 738	7 925 196	1 585 157	6 340 038
Arkansas	27 745	117 402	3 257 353	641 068	2 616 285
California	365 782	124 590	45 572 807	10 104 049	35 468 758
Colorado	54 182	118 674	6 429 993	1 331 310	5 098 683
Connecticut	34 240	124 071	4 248 218	904 474	3 343 744
Delaware	9417	121 955	1 148 389	228 172	920 217
District of Columbia	8472	132 715	1 124 394	236 200	888 194
Florida	208 088	119 582	24 883 595	4 948 813	19 934 783
Georgia	98 200	116 946	11 484 133	2 301 168	9 182 965
Hawaii	13 699	120 891	1 656 076	363 700	1 292 376
Idaho	16 735	113 755	1 903 708	385 555	1 518 153
Illinois	118 861	119 853	14 245 805	2 872 654	11 373 151
Indiana	62 282	117 322	7 307 050	1 450 408	5 856 642
Iowa	29 412	116 068	3 413 800	684 625	2 729 175
Kansas	26 726	115 976	3 099 554	617 731	2 481 823
Kentucky	41 821	118 628	4 961 122	945 036	4 016 087
Louisiana	42 552	117 158	4 985 357	990 566	3 994 791
Maine	13 264	121 403	1 610 258	321 662	1 288 596
Maryland	56 514	122 560	6 926 358	1 431 515	5 494 843
Massachusetts	67 184	126 113	8 472 822	1 817 029	6 655 793
Michigan	94 595	119 715	11 324 483	2 290 240	9 034 243
Minnesota	52 494	118 339	6 212 044	1 309 184	4 902 860
Mississippi	27 124	117 210	3 179 215	629 861	2 549 354
Missouri	57 177	117 933	6 743 085	1 334 823	5 408 263
Montana	10 298	117 968	1 214 814	250 672	964 141
Nebraska	17 691	114 817	2 031 225	412 772	1 618 452
Nevada	28 888	117 323	3 389 181	696 983	2 692 198
New Hampshire	13 524	120 808	1 633 846	339 067	1 294 779
New Jersey	86 522	124 633	10 783 488	2 347 492	8 435 996
New Mexico	19 592	118 087	2 313 513	455 745	1 857 768
New York	189 777	125 931	23 898 831	5 074 026	18 824 804
North Carolina	97 919	117 800	11 534 890	2 299 261	9 235 629
North Dakota	7099	116 165	824 677	172 576	652 101
Ohio	109 821	119 892	13 166 670	2 579 497	10 587 174
Oklahoma	36 123	117 291	4 236 945	830 542	3 406 402
Oregon	40 349	121 179	4 889 503	1 010 435	3 879 067
Pennsylvania	123 496	121 168	14 963 750	2 991 865	11 971 886
Rhode Island	10 597	122 121	1 294 141	274 823	1 019 319
South Carolina	48 310	117 915	5 696 444	1 124 197	4 572 247
South Dakota	8048	115 565	930 112	194 848	735 264
Tennessee	64 502	118 791	7 662 235	1 475 667	6 186 568
Texas	261 317	115 096	30 076 496	6 121 699	23 954 797
Utah	28 068	110 975	3 114 884	646 699	2 468 184
Vermont	6304	119 704	754 568	156 313	598 255
Virginia	65 965	119 758	7 899 865	1 620 146	6 279 719
Washington	72 073	123 369	8 891 630	1 862 573	7 029 057
West Virginia	17 139	121 923	2 089 675	379 483	1 710 191
Wisconsin	55 178	117 383	6 476 881	1 323 421	5 153 460
Wyoming	5331	117 473	626 214	129 024	497 190

Variability in state-level cost estimates was driven primarily by differences in average wage rates and demographic factors including the size of adult and unhoused populations (Table 1). For example, homelessness rates of adults with schizophrenia ranged from 2.1% in Colorado to 4.7% in Mississippi. Colorado has fewer people requiring SSDI (8.9% vs 19.6%) and a lower excess annual death rate (1.2% vs 2.0%) compared with Mississippi. Even small differences in local prevalence within a setting influenced model outcomes, including mortality rates and associated costs. In California, 3.1% of adults with schizophrenia experienced homelessness compared with 3.9% in Louisiana, corresponding to fewer additional deaths (1.2% vs 1.8%) (Table 1) and lower economic burden from shortened life expectancy ($12 331 vs $17 992) (Table 2).

## Discussion

This study provided prevalence-based estimates of the economic burden of schizophrenia in 2024 in the US from a societal perspective, disaggregated to national and state levels. Compared with prior studies, our analysis incorporated updated prevalence and cost data, accounted for residential setting, and produced state- and per-person estimates for the adult population, resulting in a higher overall burden than earlier national estimates. Direct costs made up about 20% of the total, with indirect costs predominating, including productivity losses (15%), shortened life expectancy (13%), reduced quality of life (11%), and uncompensated caregiver labor (29%). Despite conservative assumptions that include only costs clearly attributable to schizophrenia, indirect costs dominate, suggesting the true societal burden may be even greater.

These indirect costs also have direct implications for government finances. For example, 1 study^54^ estimated that lost productivity with schizophrenia results in about $30.4 billion annually in lost tax revenue. Without early intervention and ongoing management, schizophrenia often leads to acute episodes requiring emergency services, law enforcement involvement, or both.^55,56,57^ The estimated $10.1 billion in justice system-related costs may be largely preventable through targeted, sustainable, community-based interventions.^58,59,60,61^ Although expanding supportive housing services for individuals with schizophrenia may require higher initial investment, the long-term individual and societal returns may justify these costs. Although this model does not specifically evaluate such investments, the projected $2.5 million in disease-related lifetime cost for an individual diagnosed at age 18 years suggests the potential value of early and sustained interventions.^45,62^

Caregiving constituted the largest cost component in the economic burden of schizophrenia, yet it remains underrecognized in policy and planning. Many caregivers forego employment or reduce work hours, resulting in lost wages, productivity losses, and adverse health outcomes. We estimated $147.5 billion in caregiver-related costs in 2024, including unpaid labor, out-of-pocket spending, and added health care expenses.^13,14,63,64^ Policies that provide direct financial support, expand access to formal services, or create caregiver-friendly employment conditions could reduce this burden and yield economic returns through greater workforce participation and lower caregiver health care costs.

State-level estimates demonstrated geographic variation in both prevalence and costs. Where available, localized data (eg, homelessness rate) highlight differences in outcomes and per-capita costs and help identify populations where targeted services could reduce burden. Such variability reflects differences in wage structures, service delivery models, and population characteristics, although gaps in state-level data limit comprehensive evaluation of program impacts. Still, these findings support the importance of integrated data systems to support early intervention and coordinated care, particularly where targeted investment may yield cross-sector cost offsets.

Comprehensive, current data are essential to refine societal cost estimates, track intervention effectiveness, and guide resource allocation. Sensitivity analyses from this study suggest that changes in factors such as unemployment and caregiver reliance have the potential to shift overall burden, emphasizing the importance of timely localized information. Recognizing this need, Congress passed the Cost of Mental Illness Act in 2022, directing the US Department of Health and Human Services to coordinate national and local data collection to identify opportunities and target funding toward effective programs.^65^ However, the legislation did not include appropriations, and the mandated report has not been produced.

Despite the profound societal burden of schizophrenia, funding for treatment and research also remains low. Federal research funding for schizophrenia from the National Institute of Mental Health (NIMH) has declined in real terms, falling from $255 million (14% of the NIMH budget) in 2015 to $206 million (9% of the budget) in 2023.^66,67,68^ A sustained research agenda is essential to drive innovation, improve care delivery, and reduce the long-term costs associated with schizophrenia.^65^ Without these investments, existing care models remain underfunded and underdeveloped, and opportunities for groundbreaking discoveries will be missed.

### Limitations

This study has some limitations. Although sources were carefully vetted, some were older and may not reflect the current state of care. Health care cost estimates, eg, relied on MEPS data through 2015. Although costs were inflated to $2024 using a health care cost index, schizophrenia-specific changes in costs or utilization beyond inflation may not be captured. Certain inputs were unavailable for all states, populations, or settings, and some relied on small samples, limiting generalizability. For example, estimates for law enforcement encounters and supportive housing drew upon gray literature from a few states and localities.^30,41^ Sensitivity analyses varied model parameters by an arbitrary ±20% to assess their impact on total societal costs. Most inputs had minimal effect, with only 3 resulting in more than a 10% change in the projected estimate. When older studies or gray literature were used, alternative sources were reviewed to ensure inputs were comparable or more conservative (ie, lower cost or resource use).^14,15^ This estimate includes only adults with schizophrenia in the US, likely underestimating total burden. These limitations highlight the need for additional nationally and locally representative data across sectors.

## Conclusions

Results of this economic evaluation reveal that in 2024, the societal cost of schizophrenia in the US was $366.8 billion, with 80% driven by indirect costs such as unemployment, premature mortality, and caregiver burden. State-level estimates revealed variability, reflecting the influence of factors such as population size, employment, homelessness, wage rates, and cost of living. These national- and state-level estimates provide a clearer foundation for targeting care, guiding investment, and expanding early intervention to reduce the long-term impact of schizophrenia.

## Notes

Supplement 1. **eAppendix 1.** Model Framework and Sources of Data **eFigure.** Detailed Model Schematic for Estimating the Societal Costs of Schizophrenia in the United States, 2024 **eTable 1.** Representative Targeted Literature Review Topics and Search Terms **eTable 2.** Population Characteristics, Average Per Year Across 2006-2015 **eTable 3.** Average Per-Person Annual Direct Health Care Costs, Community-Dwelling Individuals With and Without Schizophrenia **eTable 4.** Estimated Excess Direct Health Care Costs, Community-Dwelling Individuals With Schizophrenia **eTable 5.** Estimated Productivity Loss, Community-Dwelling Individuals With Schizophrenia **eTable 6.** Population Characteristics, Average Per Year Across 2006-2014 (*ICD-9* 295) **eTable 7.** Average Per-Person Annual Direct Health Care Costs, Community-Dwelling Individuals With and Without Schizophrenia (*ICD-9* 295) **eTable 8.** Estimated Excess Direct Health Care Costs, Community-Dwelling Individuals With Schizophrenia (*ICD-9* 295) **eTable 9.** Estimated Productivity Loss, Community-Dwelling Individuals With Schizophrenia (*ICD-9* 295) **eAppendix 2.** Model Parameters **eTable 10.** National and State Model Input Parameters: Prevalence **eTable 11.** National and State Model Input Parameters: Health Care Costs **eTable 12.** National and State Model Input Parameters: Social Security Disability Benefits **eTable 13.** National and State Model Input Parameters: Supportive Housing and Homelessness **eTable 14.** National and State Model Input Parameters: Justice System Interactions **eTable 15.** National and State Model Input Parameters: Reduced Quality of Life **eTable 16.** National and State Model Input Parameters: Shortened Life Expectancy **eTable 17.** National and State Model Input Parameters: Nonemployment and Reduced Wages **eTable 18.** National and State Model Input Parameters: Caregiver Unpaid Wages and Other Impacts **eTable 19.** National and State Model Input Parameters: Lifetime Cost of Schizophrenia Scenario
eReferences.

Supplement 2.
Data Sharing Statement.
