# Supplementary material for: National and State Societal Costs of Schizophrenia in the US in 2024
Source: JAMA Psychiatry. 2026 Jan 28;83(4):341–52. doi: 10.1001/jamapsychiatry.2025.4383 (PMC12853289; doi:10.1001/jamapsychiatry.2025.4383)
Supplement: Supplement 1. — eAppendix 1. Model Framework and Sources of Data eFigure. Detailed Model Schematic for Estimating the Societal Costs of Schizophrenia in the United States, 2024 eTable 1. Representative Targeted Literature Review Topics and Search Terms eTable 2. Population Characteristics, Average Per Year Across 2006-2015 eTable 3. Average Per-Person Annual Direct Health Care Costs, Community-Dwelling Individuals With and Without Schizophrenia eTable 4. Estimated Excess Direct Health Care Costs, Community-Dwelling Individuals With Schizophrenia eTable 5. Estimated Productivity Loss, Community-Dwelling Individuals With Schizophrenia eTable 6. Population Characteristics, Average Per Year Across 2006-2014 (ICD-9 295) eTable 7. Average Per-Person Annual Direct Health Care Costs, Community-Dwelling Individuals With and Without Schizophrenia (ICD-9 295) eTable 8. Estimated Excess Direct Health Care Costs, Community-Dwelling Individuals With Schizophrenia (ICD-9 295) eTable 9. Estimated Productivity Loss, Community-Dwelling Individuals With Schizophrenia (ICD-9 295) eAppendix 2. Model Parameters eTable 10. National and State Model Input Parameters: Prevalence eTable 11. National and State Model Input Parameters: Health Care Costs eTable 12. National and State Model Input Parameters: Social Security Disability Benefits eTable 13. National and State Model Input Parameters: Supportive Housing and Homelessness eTable 14. National and State Model Input Parameters: Justice System Interactions eTable 15. National and State Model Input Parameters: Reduced Quality of Life eTable 16. National and State Model Input Parameters: Shortened Life Expectancy eTable 17. National and State Model Input Parameters: Nonemployment and Reduced Wages eTable 18. National and State Model Input Parameters: Caregiver Unpaid Wages and Other Impacts eTable 19. National and State Model Input Parameters: Lifetime Cost of Schizophrenia Scenario eReferences. [file jamapsychiatry-e254383-s001.pdf]

Supplementary Online Content

Krasa HB, Baumgardner JR, Brewer IP, et al. National and state societal costs of schizophrenia in the US in 2024. *JAMA Psychiatry.* Published online January 28, 2026. doi:10.1001/jamapsychiatry.2025.4383

eAppendix 1. Model Framework and Sources of Data

eFigure. Detailed Model Schematic for Estimating the Societal Costs of Schizophrenia in the United States, 2024

eTable 1. Representative Targeted Literature Review Topics and Search Terms

eTable 2. Population Characteristics, Average Per Year Across 2006-2015

eTable 3. Average Per-Person Annual Direct Health Care Costs, Community-Dwelling Individuals With and Without Schizophrenia

eTable 4. Estimated Excess Direct Health Care Costs, Community-Dwelling Individuals With Schizophrenia

eTable 5. Estimated Productivity Loss, Community-Dwelling Individuals With Schizophrenia

eTable 6. Population Characteristics, Average Per Year Across 2006-2014 (*ICD-9* 295)

eTable 7. Average Per-Person Annual Direct Health Care Costs, Community-Dwelling Individuals With and Without Schizophrenia (*ICD-9* 295)

eTable 8. Estimated Excess Direct Health Care Costs, Community-Dwelling Individuals With Schizophrenia (*ICD-9* 295)

eTable 9. Estimated Productivity Loss, Community-Dwelling Individuals With Schizophrenia (*ICD-9* 295)

eAppendix 2. Model Parameters

eTable 10. National and State Model Input Parameters: Prevalence

eTable 11. National and State Model Input Parameters: Health Care Costs

eTable 12. National and State Model Input Parameters: Social Security Disability Benefits

eTable 13. National and State Model Input Parameters: Supportive Housing and Homelessness

eTable 14. National and State Model Input Parameters: Justice System Interactions

eTable 15. National and State Model Input Parameters: Reduced Quality of Life

eTable 16. National and State Model Input Parameters: Shortened Life Expectancy

© 2026 Krasa HB et al. *JAMA Psychiatry*.

eTable 17. National and State Model Input Parameters: Nonemployment and Reduced Wages

eTable 18. National and State Model Input Parameters: Caregiver Unpaid Wages and Other Impacts

eTable 19. National and State Model Input Parameters: Lifetime Cost of Schizophrenia Scenario

eReferences.

This supplementary material has been provided by the authors to give readers additional information about their work.

© 2026 Krasa HB et al. *JAMA Psychiatry*.

eAppendix 1. Model Framework and Sources of Data

The societal costs of schizophrenia spectrum disorders within the United States (US) are modeled using a prevalence-based, input-output framework spanning seven domains grouped into two major categories, direct medical, direct non-medical, and indirect costs for the calendar year 2024. Costs are estimated using an excess cost approach for the adult population of the US, adjust for state-level population variations.

Direct cost domains included health care, social security disability benefits, supportive housing and homelessness, and justice system interactions. Indirect cost domains for those living with schizophrenia included costs resulting from reduced quality of life, non-employment and reduced wages from underemployment, and shortened life expectancy. Indirect cost domains for caregivers of those living with schizophrenia include caregiving related unpaid wages and other caregiver impacts.

The model first estimates prevalence of schizophrenia spectrum disorders by setting (community-dwelling, independent household; community dwelling, structured residence; long-term care/skilled nursing facility; homeless or unhoused; and incarcerated). Structured and independent living settings were combined as one community dwelling setting for some estimates. Domain specific costs are calculated and aggregated at the national or state level. Estimates represent costs incurred in the year 2024.

The full workflow appears in the eFigure, which depicts the sequence from cost inputs, $2024 adjustments, and application by adult residential setting to calculation of excess costs by category, domains, and totals. Specific model input parameters, parameter sources, and calculation methods are listed in eAppendix 2 (eTable 10 to eTable 18).

© 2026 Krasa HB et al. *JAMA Psychiatry*.

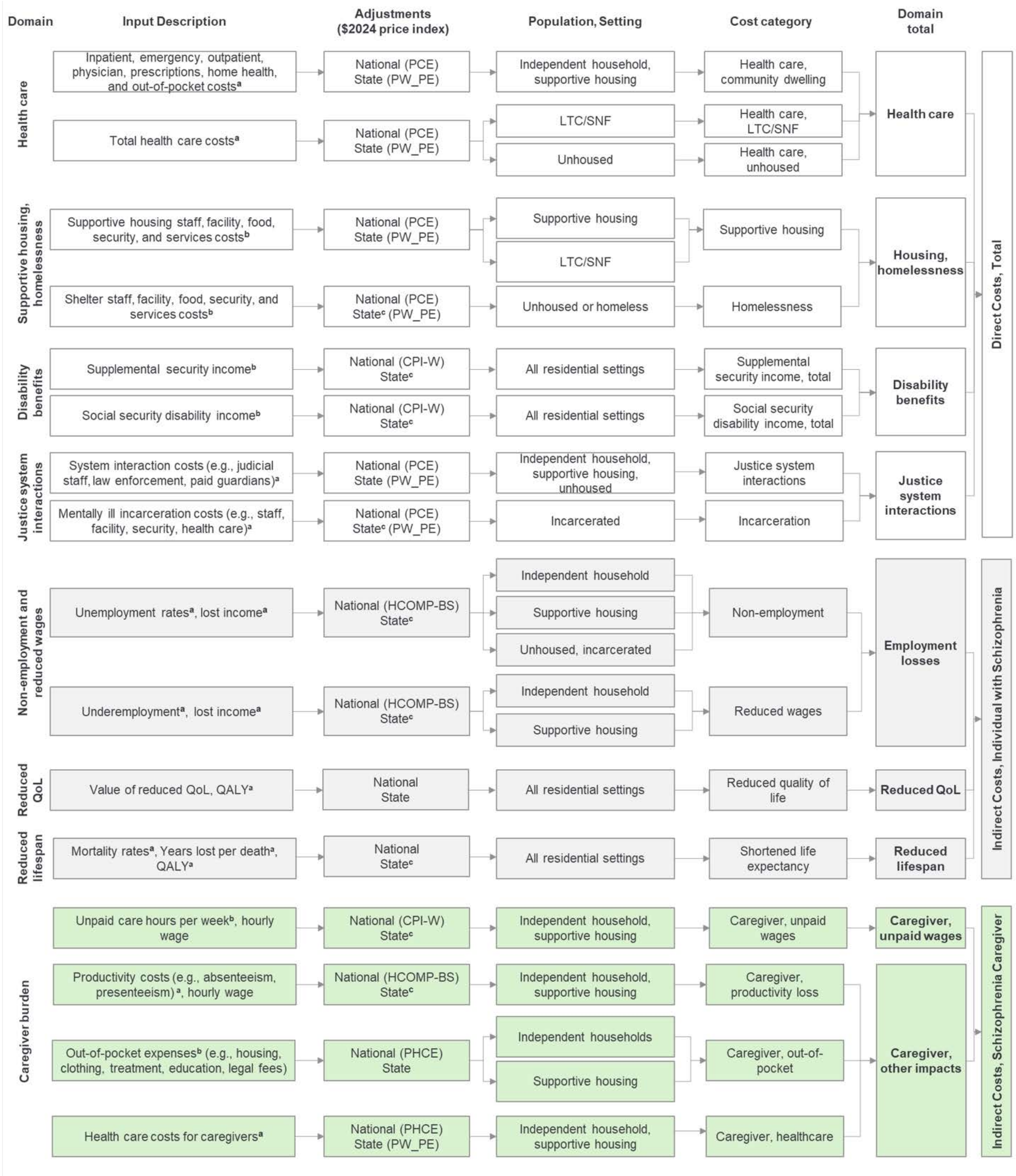

eFigure. Detailed Model Schematic for Estimating the Societal Costs of Schizophrenia in the United States, 2024

Incremental or excess costs were estimated as **(a)** per-person differentials by comparing adults with schizophrenia with adults without the condition or **(b)** by applying general-population unit costs or rates to the additional number of adults incurring costs for the cost category **(c)** marks inputs with state-specific data, either as direct input or adjusted using ratio of state to national data; otherwise, state values are derived from national inputs. All state estimates are based on the state-specific population of adults. Price indices applied to model inputs for National and State estimates are noted in the “Adjustments” column in parentheses. Abbreviations: LTC, long-term care; SNF, skilled nursing facility; QoL, quality of life; QALY, quality adjusted life year; PHCE, Personal Health Care Expenditure Index; PW, physician wage; PE, practice expense; PW_PE, Center for Medicare and Medicaid Services physician wage (PW) and practice expense (PE) Geographic Practice Cost Indices; CPI_W, Consumer Price Index – Urban Wage Earners and Clerical Workers; HCOMP-BS, Business Sector: Hourly Compensation for All Workers.

© 2026 Krasa HB et al. *JAMA Psychiatry*.

S1.1 Model Parameter Adjustments (National and State-Level)

S1.1.1 National Parameter Adjustments

All abstracted national cost parameters were inflated to $2024 prior to model entry using one of four indices:

- **Personal Health Care Expenditure Index (PHCE):** for health care expenditures, long-term care facility costs, and caregiver out-of-pocket expenses.[1]
- **Consumer Price Index – Urban Wage Earners and Clerical Workers (CPI-W):** for social security disability benefit costs.[2]
- **Business Sector: Hourly Compensation for All Workers (HCOMP-BS):** for wage related indirect costs for those with schizophrenia and their caregivers.[3]
- **Personal Consumption Expenditures (PCE) Price Index:** for judicial system, supportive housing, and homelessness costs.[4]

S1.1.2 State Parameter Adjustments

State-specific values were used where available. State-specific data informed the number of homeless adults with schizophrenia in each state, the number receiving social security disability income (SSDI) and social security income (SSI) in each state, general mortality rate in each state, the number of adults with schizophrenia in each state.

For others state model parameters, the following approaches were used to adjust national estimates for state-level estimates:

- **Physician Wage (PW) and Practice Expense (PE) (health care costs):** National estimates were adjusted using a combination of Center for Medicare and Medicaid Services (CMS) PW and PE Geographic Practice Cost Indices (GPCIs).[5] A combined PW_PE values for each state was constructed in four steps:
    1. Match US Census Zip Code Tabulation Area (ZCTA) census cost to USPS Zip codes for all zip codes with non-zero populations using exact of nearest neighbor spatial matching.
    2. Estimate the US 2020 adult population (18+) within each ZCTA.
- Aggregate ZCTA population estimates for CMS Medicare administrative contractor (MAC) geographic jurisdiction.
    3. Calculate a MAC-population weighted value for each state where: $PW\_PE = (PW + PE)/2$.
    4. PW_PE was applied to the national health care cost estimates such as long-term care facility, homelessness remediation, structured residence, direct health care, law enforcement contact, incarceration, and caregiver health care costs.
- **Crude Wage Ratio (CWR) (productivity costs):** For state-specific productivity costs, a wage index based on each state's average wage relative to the national average was developed and normalized, so a national, population-weighted average of state indices equaled one.[6] CWR values for each state were constructed in three steps:
    5. Calculate the ratio of the average hourly wage for all employed persons within a state to the national average (CWR)

© 2026 Krasa HB et al. *JAMA Psychiatry*.

6. Estimate the average CWR across all states including DC
7. Normalized all state CWR so the national average equals 1: State CWR/Average CWR

- **State employment rate relative to the national average:** For state-specific employment rates, the state's estimated employment rate relative to the national average was calculated by taking the state employment rate and dividing by the national employment rate.
- **State population percentage:** The percentage of the estimated 2024 US adult population who reside in each state was used to adjust the national values for the general population.
- **State percentage of those receiving social security disability income (SSDI) out of state's total population:** The ratio of those receiving SSDI with schizophrenia to the state's total population. It is further adjusted to arrive at a proxy measure of each state's schizophrenia prevalence relative to the national prevalence.
- **State homelessness percentage of national total:** The percentage of the nation's total homeless persons who reside in each state. Assuming the risk of homelessness given schizophrenia is constant across states, the number of homeless with schizophrenia for each state = risk of homelessness given schizophrenia x number with schizophrenia in each state.
- **State ratio of those with SSDI to those who are homeless normalized to the national level:** A normalization of the ratio of those receiving SSDI with schizophrenia in the state to the total number of homeless persons in the state to the national level. These normalized values are used to adjust schizophrenia prevalence among the homeless for each state.
- **State ratio of those who receive SSDI who have schizophrenia relative to national ratio:** This is the percentage of those who receive SSDI with schizophrenia in the state normalized to the national percentage of those receiving SSDI with schizophrenia.

S1.2 Targeted Literature Reviews

Targeted literature reviews of peer-reviewed and grey literature were conducted in two stages: 1) initial targeted literature review conducted in 2020; 2) supplemental targeted literature reviews conducted in 2024.

Questions guiding the literature reviews were:

- What are the direct health care costs incurred by patients with schizophrenia spectrum disorders?
- What are the indirect costs for patients with schizophrenia spectrum disorders?
- What are caregiver costs including absenteeism, presenteeism, unpaid labor, health care costs, and other out of pocket (OOP) expenses?
- What are the direct non-health care costs associated with schizophrenia spectrum disorders, such as social security disability income (SSDI), homelessness, justice system interactions, and research?
- How do costs of schizophrenia spectrum disorders vary across settings and regions?

The following were searched: 1) peer-reviewed literature using Google Scholar, PubMed, and conference proceedings, 2) grey-literature including white papers by patient advocacy or professional societies, and 3) government provided, publicly available data and publications such as the US Department of Housing and Urban Development, Coalition of State Governments, Bureau of Justice Statistics, and National Sheriff's Association.

© 2026 Krasa HB et al. *JAMA Psychiatry*.

Model parameters identified from the reviews for national level data identified cost heterogeneity in certain parameters across states. To adjust for state-level costs, an additional targeted literature review was conducted to identify state-level values where available.

eTable 1 provides an example of topics and associated search terms from searches of PubMed that included publications from 2000-2024. Full literature review outputs are available from the authors.

eTable 1: Representative Targeted Literature Review Topics and Search Terms

Topic	Search terms
Overall economic burden	(schizophrenia [Title/Abstract] OR schizoaffective [Title/Abstract] OR schizophreniform [Title/Abstract] OR delusional [Title/Abstract]) AND (economic burden [Title/Abstract] OR cost [Title/Abstract] OR spending [Title/Abstract]) AND (United States [Title/Abstract])
Justice system costs	(schizophrenia [Title/Abstract]) AND (criminal justice [Title/Abstract] OR incarceration [Title/Abstract] OR prisoner [Title/Abstract] OR legal [Title/Abstract] OR judicial [Title/Abstract]) AND (cost [Title/Abstract])
Adherence costs	(schizophrenia [Title/Abstract] OR schizoaffective [Title/Abstract] OR schizophreniform [Title/Abstract] OR delusional [Title/Abstract]) AND (adherence [Title/Abstract]) AND (cost [Title/Abstract])
Housing costs	(schizophrenia OR schizoaffective OR delusional OR psychosis) AND (housing [Title/Abstract] OR homeless [Title/Abstract]) AND (cost [Title/Abstract] OR burden [Title/Abstract] OR spending [Title/Abstract])
Caregiver burden	(schizophrenia [Title/Abstract]) AND (caregiver [Title/Abstract]) AND (cost [Title/Abstract] OR burden [Title/Abstract] OR financial [Title/Abstract])
Social determinants and cost	(schizophrenia [Title/Abstract]) AND (race [Title/Abstract] OR ethnicity [Title/Abstract] OR disparity [Title/Abstract] OR social determinant [Title/Abstract]) AND (cost [Title/Abstract] OR economic burden [Title/Abstract] OR spending [Title/Abstract])
Indirect costs	(schizophrenia [Title/Abstract] OR schizoaffective [Title/Abstract] OR schizophreniform [Title/Abstract] OR delusional [Title/Abstract]) AND (indirect [Title/Abstract] OR productivity [Title/Abstract] OR disability [Title/Abstract] OR sick days [Title/Abstract] OR unemployment [Title/Abstract] OR underemployment [Title/Abstract]) AND (cost [Title/Abstract] OR economic burden [Title/Abstract])
Caregiver burden	(schizophrenia [Title/Abstract] OR schizoaffective [Title/Abstract] OR schizophreniform [Title/Abstract] OR delusional [Title/Abstract] OR psychosis [Title/Abstract]) AND (caregiver [Title/Abstract] OR family [Title/Abstract]) AND (cost [Title/Abstract] OR economic [Title/Abstract])
Housing costs	((housing OR homeless OR group home OR assisted living OR residential) AND (schizophrenia [Title/Abstract] OR schizoaffective [Title/Abstract] OR psychosis [Title/Abstract] OR delusion [Title/Abstract] OR hallucination [Title/Abstract] OR schizophreniform [Title/Abstract])) AND (cost [Title/Abstract])

S1.3 Medical Expenditure Panel Survey Empirical Analysis

To estimate direct health care costs and productivity loss in community-dwelling individuals with schizophrenia spectrum disorders, an analysis of the Medical Expenditure Panel Survey (MEPS) was

© 2026 Krasa HB et al. *JAMA Psychiatry*.

conducted in 2020.[7] MEPS is a set of large-scale surveys administered annually to a sample of the US civilian noninstitutionalized population and collected information from individuals and families, their medical providers, and employers. The information collected includes the type, usage frequency, cost, and method of payment for various medical services, detailed insurance information, access to care, satisfaction with care, employment information, and demographic characteristics.

MEPS was selected since it is the most complete source of data on health care utilization and health insurance combined with individual and family-level characteristics for the US community population. The participating individuals in the MEPS are a subsample of households in the US with identifiable addresses, from which nationally representative estimates are derived using survey weights. However, as the MEPS does not survey individuals who are in assisted living facilities or other institutions, homeless, or incarcerated, the results of this analysis are considered applicable only to community dwelling persons (i.e., those in independent households or structured housing).

S1.3.1 MEPS Analysis Methods

Data Description

A pooled sample from 2006 to 2015 using the full-year consolidated data and medical conditions file was used. Data from 2016 onward was excluded because data specific to schizophrenia spectrum disorders was no longer collected. The person-specific weights provided by MEPS were used for all analyses to obtain national estimates.

Analytic Sample

The analytic sample was identified using the Agency for Healthcare Research and Quality's (AHRQ) clinical classification system (CCS) which aggregates conditions into mutually exclusive categories using multiple International Classification of Diseases, 9th edition (ICD-9) codes.[8,9] MEPS disease categories encompass multiple ICD-9 codes rather than reporting the number of individuals with specific codes to protect the identity of individuals with rare conditions. For the years 2006-2015, ICD-9 codes representing schizophrenia spectrum disorders were mapped to the MEPS clinical category 659 "schizophrenic and psychotic disorders", which includes ICD-9 codes 293.81. 293.82, 295.XX, 297.X, and 298.X identified cases (i.e., those with a schizophrenia spectrum disorder).[10] Major codes 297 and 298 refer to delusional disorders and other non-organic psychoses, respectively. Following prior work,[11] this CCS category was chosen for base case analysis to address limitations in MEPS interview-based reporting and include individuals with related interim diagnoses.

People with a diagnosis under this MEPS category were matched to people without a diagnosis in this category and included in the base case analysis. Coarsened Exact Matching identified a set of controls balanced across race, gender, age, health care plan, and geographic region.[12]

For the years 2006-2015, the MEPS specifically reported on "schizophrenic disorders" (ICD-9: 295.XX), which includes schizophrenia, schizophreniform disorder, schizoaffective disorder, and other/unspecified schizophrenia. A sensitivity analysis of these subsamples and matched controls was also conducted.

Direct Health Care and Productivity Loss Cost Estimates

All-cause health care and productivity costs were calculated and summed up. Direct medical costs include inpatient stays, outpatient visits, office-based physician visits, emergency room visits, home health care visits, and prescription medications. Dental and vision costs were excluded.

Unemployment, the average income of those with and without schizophrenia, and the number of hours absent from work were used to calculate the indirect costs of productivity loss and unemployment. Productivity loss was estimated for those individuals with employment and missed time from work. The value of productivity loss was estimated using the human capital (HC) approach, where time away from

work is valued at the individual's hourly wage to represent his or her marginal product. Any time away from work, regardless of the duration, will result in a loss of that marginal product. Therefore, missed time is valued at the hourly wage for as long as the absence from work lasts. Specifically, for every individual who is not employed in each quarter, a value for that job loss was assigned as follows:

(1) HC_value = (1-employed) x job_loss_value + employed x missed_days x daily_wage

The value of days missed was approximated at the individual's hourly wage, using data on wages by industry. Individuals were assigned an hourly wage based on his or her industry of occupation with fringe benefits approximated by a factor of 1.26.[13] At the time this analysis was conducted, wages were adjusted to $2020, using the Consumer Price Index prior to analysis.[14]

Descriptive statistics were summarized for all study variables and were used to inform the regression analyses. Continuous variables were summarized using the mean and standard deviation, while binary variables were summarized as frequencies and percentages.

Regression analyses were then conducted to estimate *excess* health care costs for community dwelling individuals with schizophrenia compared to those without, controlling for confounding factors and unbalanced matching. Excess costs attributable to schizophrenia were calculated as the difference in costs between those with schizophrenia and those without. MEPS analysis results are presented in $2020 in this section. Results were adjusted to $2024 when included in the model parameters (see section eAppendix 2).

S1.3.2 MEPS Analysis Results

Characteristics of Community-Dwelling Schizophrenia and Non-Schizophrenia Populations

Analysis of MEPS data from 2006-2015 of 348,364 households per year, on average, represented an estimated 309,136,995 individuals in the US population. Using the person-specific weights provided in MEPS to estimate, 901,800 individuals were identified (unweighted frequency = 1,038) with schizophrenia in a given year. Characteristics of the total population, those with schizophrenia, and those without schizophrenia are provided in eTable 2. Compared to individuals without schizophrenia, those with schizophrenia were older (mean age: 43.94 vs. 37.09 years), more likely to identify as male (57.96% vs. 48.95%), had lower employment rates (37.27% vs. 63.61%), and reported lower annual income among those employed ($24,766.18 vs. $45,266.77).

eTable 2: Population Characteristics, Average per Year Across 2006-2015

Characteristic	Total population	Schizophrenia population[a]	Non-schizophrenia population
**MEPS sample size, No.**	349,405	1,038	348,364
**MEPS sample size, Weighted No.**	310,038,796	901,800	309,136,995
**Age, mean**	37.11	43.94	37.09
**Age range, No. (%)**			
18-30	54,889,268 (17.70%)	144,559 (16.03%)	54,745,070 (17.71%)
31-64	136,851,125 (44.14%)	445,128 (49.36%)	135,896,623 (43.96%)
65 and over	41,576,203 (13.41%)	120,661 (13.38%)	41,486,185 (13.42%)
**Sex, No. (%)**			

© 2026 Krasa HB et al. *JAMA Psychiatry*.

Characteristic	Total population	Schizophrenia population[a]	Non-schizophrenia population
Female	158,191,095 (51.02%)	379,081 (42.04%)	157,811,345 (51.05%)
Male	151,847,701 (48.98%)	522,719 (57.96%)	151,325,650 (48.95%)
**Race and Ethnicity, No. (%)**			
Hispanic or Latino/a	51,249,413 (16.53%)	113,537 (12.59%)	51,131,259 (16.54%)
Non-Hispanic White	197,246,682 (63.62%)	561,100 (62.22%)	196,672,956 (63.62%)
Non-Hispanic Black	37,607,706 (12.13%)	182,975 (20.29%)	37,405,576 (12.10%)
Non-Hispanic Asian	15,067,885 (4.86%)	14,519 (1.61%)	15,054,972 (4.87%)
Non-Hispanic Other or Multiple Races	8,898,113 (2.87%)	29,579 (3.28%)	8,872,232 (2.87%)
**Geographic Regions, No. (%)**			
Northeast	54,969,879 (17.73%)	198,667 (22.03%)	54,779,076 (17.72%)
Midwest	66,317,298 (21.39%)	167,104 (18.53%)	66,124,403 (21.39%)
South	113,908,254 (36.74%)	284,067 (31.50%)	113,638,759 (36.76%)
West	72,239,039 (23.30%)	203,446 (22.56%)	72,028,920 (23.30%)
Unidentified	2,604,326 (0.84%)	48,517 (5.38%)	2,565,837 (0.83%)
**Employment**			
Employed, No. (%)	196,967,069 (63.53%)	336,081 (37.27%)	196,630,988 (63.61%)
Overall population total income, mean $2020	$32,056.52	$18,311.48	$32,096.62
Working population total income, mean $2020	$45,231.79	$24,766.18	$45,266.77

[a] Schizophrenia population includes ICD-9 295.xx, 297.1, and 298.x. Sensitivity analysis with ICD-9 295 only presented in eTable 7.
Abbreviation: MEPS, Medical Expenditure Panel Survey.

Average Direct Health Care Costs, Community-Dwelling Populations

Average direct health care costs were calculated for individuals with and without schizophrenia for the year 2020. These estimates are not risk-adjusted. Individuals with schizophrenia had mean annual direct health care costs of $19,768, compared to $6,064 for those without schizophrenia (eTable 3). The differences in costs were primarily driven by prescription medications, inpatient hospital stays, and office visits.

eTable 3: Average Per-Person Annual Direct Health Care Costs, Community-Dwelling Individuals with and without Schizophrenia

Cost category	Schizophrenia[a] n=901,800 mean (SD), $2020	Non-schizophrenia n=309,136,995 mean (SD), $2020
**Total direct health care costs, per person annually**	$19,768.08 ($948.41)	$6,064.45 ($63.10)

© 2026 Krasa HB et al. *JAMA Psychiatry*.

Cost category	Schizophrenia[a] n=901,800 mean (SD), $2020	Non-schizophrenia n=309,136,995 mean (SD), $2020
**Direct health care cost type**		
Inpatient hospital stays	$5,173.46 ($628.62)	$1,524.45 ($29.04)
Outpatient visits	$1,588.46 ($179.99)	$935.37 ($25.75)
Office-based physician visits	$3,384.65 ($285.09)	$1,263.63 ($13.07)
Emergency room visits	$473.18 ($50.89)	$212.77 ($3.39)
Home health care visits	$2,028.37 ($477.43)	$204.24 ($10.97)
Prescription medications	$5,833.47 ($258.33)	$1,150.39 ($18.47)
Out-of-pocket	$1,286.49 ($123.43)	$773.59 ($9.18)

[a]Schizophrenia population includes ICD-9 295.xx, 297.1, and 298.x. Sensitivity analysis with ICD-9 295 only presented in eTable 7

Analysis of total health care expenditures, broken down by type of payer that incurs the costs showed total annual health care expenditures for individuals with schizophrenia summed to nearly $16.4 billion across all payer types, with Medicare incurring the largest portion of costs ($6,549,527,958; 40%), followed by Medicaid ($5,351,435,228; 33%), other or unknown ($2,653,212,493; 21%), and private/commercial insurers ($1,070,896,249; 7%).

There were variations in health care costs across race and ethnicity. People with schizophrenia (ICD-9 295.xx, 297.1, and 298.x) who identified as non-Hispanic other or multiple races incurred the highest average per-person annual direct health care costs ($22,643), followed by non-Hispanic white ($21,803), Hispanic or Latino/a ($16,409), non-Hispanic Black ($16,044) and non-Hispanic Asian ($8,489).

Estimated Excess Direct Health Care Costs

eTable 4 presents estimates from a sample of 1,033 successful case/control matches. All matching criteria resulted in exact matches except the age criteria, with mean age for the schizophrenia population at 44.12 years and for matched controls at 44.17.

In the matched sample, the per-person total excess direct health care cost was $11,548.91 in 2020 for community-dwelling individuals with schizophrenia. These costs were primarily driven by prescription medication and inpatient costs. Total direct excess health care costs attributable to schizophrenia amounted to $10.44 billion in 2020.

eTable 4: Estimated Excess Direct Health Care Costs, Community-Dwelling Individuals with Schizophrenia[a]

Cost category	Excess per person cost mean (SE), $2020	Excess total annual cost $2020
**Total excess costs, direct health care**	$11,548.95 ($754.5)	$10,414,843,060
**Excess direct health care cost type**		
**Inpatient hospital stays**	$2,162.18 ($322.10)	$1,949,851,452
**Outpatient visits**	$831.58 ($268.80)	$749,920,775
**Office-based visits**	$2,005.92 ($223.90)	$1,808,942,162
**Emergency room visits**	$162.62 ($76.45)	$146,652,876

© 2026 Krasa HB et al. *JAMA Psychiatry*.

Cost category	Excess per person cost mean (SE), $2020	Excess total annual cost $2020
**Home health care visits**	$1,512.01 ($339.80)	$1,363,527,129
**Prescription medications**	$4,559.58 ($299.00)	$4,111,832,651
**Out-of-pocket**	$315.16 ($97.88)	$284,211,741

[a]Schizophrenia population includes ICD-9 295.xx, 297.1, and 298.x. Sensitivity analysis with ICD-9 295 only presented in eTable 8.

Estimated Excess Productivity Loss

Estimated annual lost income due to unemployment was over $9.3 billion and reduced wages among the employed were $4.2 billion in 2020. For those employed, there was no loss of income due to absenteeism. The total productivity loss costs summed to over $13.5 billion, for community-dwelling individuals with schizophrenia (eTable 5).

eTable 5: Estimated Productivity Loss, Community-Dwelling Individuals with Schizophrenia[a]

Productivity category	Average excess per person cost, $2020	Average annual excess total cost, $2020
**Total costs, unemployment and productivity loss**	$51,741.73	$13,544,391,788
**Lost income due to unemployment**	$39,017.15	$9,267,903,497
**Reduced wages among employed**	$12,727.75	$4,277,553,550
**Loss of income due to work absenteeism**	- $3.17	- $1,056,259

[a]Schizophrenia population includes ICD-9 295.xx, 297.1, and 298.x. Sensitivity analysis with ICD-9 295 only presented in eTable 9.

MEPS Sensitivity Analyses

Overall, the results from the sensitivity analysis limited to MEPS respondents with ICD-9 code 295 ("schizophrenic disorders") did not meaningfully differ from the main analysis. Sensitivity analysis identified 648,425 individuals (unweighted frequency = 704) or 72% of the population included in the main analysis.

Characteristics of the total population, those with schizophrenia, and those without schizophrenia from this sensitivity analysis are shown in the eTable 6.

eTable 6. Population Characteristics, Average per Year Across 2006-2014 (ICD-9 295)

Characteristic	Total population	Schizophrenia population	Non-schizophrenia population
MEPS Weighted No.	308,773,856	648,425	308,125,431
Age, mean	37.02	40.87	37.01
Age, No. (%)			
18-30	50,595,684 (16.39%)	111,159 (17.14%)	50,484,525 (16.38%)
31-64	136,447,167 (44.19%)	432,283 (66.67%)	136,014,884 (44.14%)

© 2026 Krasa HB et al. *JAMA Psychiatry*.

Characteristic	Total population	Schizophrenia population	Non-schizophrenia population
65 and over	40,881,659 (13.24%)	30,877 (4.76%)	40,850,781 (13.26%)
Sex, No. (%)			
Female	157,520,983 (51.02%)	237,229 (36.59%)	157,273,642 (51.04%)
Male	151,252,873 (48.99%)	411,196 (63.41%)	150,851,789 (48.96%)
Race and Ethnicity, No. (%)			
Hispanic or Latino/a	50,595,230 (16.39%)	84,577 (13.04%)	50,510,341 (16.39%)
Non-Hispanic White	197,570,561 (63.99%)	375,899 (57.97%)	197,194,101 (64.00%)
Non-Hispanic Black	37,392,984 (12.11%)	159,757 (24.64%)	37,234,396 (12.08%)
Non-Hispanic Asian	14,767,311 (4.78%)	9,084 (1.40%)	14,757,911 (4.79%)
Non-Hispanic Other or Multiple Races	8,447,770 (2.74%)	19,108 (2.95%)	8,428,682 (2.74%)
Geographic Regions, No. (%)			
Northeast	54,881,864 (17.77%)	142,407 (21.96%)	54,740,119 (17.77%)
Midwest	66,200,959 (21.44%)	129,748 (20.01%)	66,070,984 (21.44%)
South	113,268,139 (36.68%)	205,699 (31.72%)	113,061,656 (36.69%)
West	71,835,805 (23.26%)	145,571 (22.45%)	71,690,105 (23.27%)
Unidentified	2,587,089 (0.84%)	25,000 (3.86%)	2,538,430 (0.83%)
Employment			
Employed, No. (%)	183,138,605 (59.31%)	170,073 (26.21%)	182,968,531 (59.38%)
Overall population total income, mean $2020	$31,906.44	$13,742.92	$31,943.83
Working population total income, mean $2020	$47,548.04	$19,780.60	$45,573.85

Abbreviation: MEPS, Medical Expenditure Panel Survey.

Sensitivity analysis of per person, per year direct health care costs were similar ($17,292.63, ICD-9 295) to the main analysis population with schizophrenia ($19,768.08) with marginally lower costs for health care visits and higher costs for prescription medications (eTable 7).

eTable 7: Average Per-Person Annual Direct Health Care Costs, Community-Dwelling Individuals with and without Schizophrenia (ICD-9 295)

Cost category	Schizophrenia n = 648,425 mean (SD), $2020	Non-schizophrenia n = 308,125,431 mean (SD), $2020
**Total direct health care costs, per person annually**	$17,292.63 ($940.86)	$5,592.75 ($61.76)
Direct health care cost type		
**Inpatient hospital stays**	$4,075.98 ($602.09)	$1,436.19 ($30.06)

© 2026 Krasa HB et al. *JAMA Psychiatry*.

Cost category	Schizophrenia<br>n = 648,425<br>mean (SD), $2020	Non-schizophrenia<br>n = 308,125,431<br>mean (SD), $2020
Outpatient visits	$1,089.57 ($124.62)	$802.72 ($23.62)
Office-based physician visits	$3,677.11 ($370.28)	$1,416.79 ($15.90)
Emergency room visits	$322.81 ($33.93)	$196.68 ($3.29)
Home health care visits	$1,698.53 ($594.51)	$185.49 ($10.53)
Prescription medications	$7,116.85 ($304.46)	$1282.14 ($21.40)
Out-of-pocket	$1,409.91 ($83.75)	$892.75 ($11.16)

Estimated excess direct health care costs per person per year were also similar in the sensitivity analysis population ($10,387.40 vs. $11,548.95) compared to the main analysis population (eTable 8).

eTable 8: Estimated Excess Direct Health Care Costs, Community-Dwelling Individuals with Schizophrenia (ICD-9 295)

Cost category	Excess per person annual costs mean (SE), $2020	Excess total annual costs $2020
Total excess costs, direct health care	$10,387.40 ($843.00)	$6,735,449,845
Excess direct health care cost type		
Inpatient hospital stays	$1,955.60 ($475.20)	$1,268,059,930
Outpatient visits	$522.00 ($337.40)	$338,477,850
Office-based visits	$1,960.40 ($336.60)	$1,271,172,370
Emergency room visits	$149.70 ($43.25)	$97,069,223
Home health care visits	$1,416.40 ($245.40)	$918,429,170
Prescription medications	$5,267.30 ($296.40)	$3,415,449,003
Out-of-pocket	$435.50 ($143.50)	$282,389,088

Excess per person costs from productivity loss were marginally higher in sensitivity analysis populations ($54,129.80 vs. $51,741.73) compared to the main analysis population (eTable 9).

eTable 9: Estimated Productivity Loss, Community-Dwelling Individuals with Schizophrenia (ICD-9 295)

Productivity category	Average excess cost per person, $2020	Total excess annual cost, $2020
Total costs, unemployment and productivity loss	$54,129.80	$10,942,893,446
Lost income due to unemployment	$39,017.15	$8,391,908,994
Reduced wages among employed	$15,112.65	$2,570,253,723
Loss of income due to work absenteeism	- $113.30	- $19,269,271

© 2026 Krasa HB et al. *JAMA Psychiatry*.

S1.3.3 MEPS Analysis Conclusions

Average direct health care costs among community-dwelling individuals with schizophrenia were $19,768.08 per person, per year in 2020. Prescription medications and inpatient stay costs were the two largest components of direct health care costs. Total annual health care costs among community-dwelling individuals with schizophrenia in the MEPS data amounted to $16.4 billion, with Medicare incurring nearly 40% of those expenditures. Community-dwelling individuals with schizophrenia incurred $11,548.95 more on average in direct health care costs than those without schizophrenia. Total direct excess health care costs attributable to schizophrenia amounted to $10.44 billion in 2020 in the MEPS population.

Excess unemployment and productivity loss costs (including lost income due to unemployment, reduced wages among the employed, and loss of income due to absenteeism from work) for community-dwelling individuals with schizophrenia was estimated at $51,741.73 per person annually. Total excess unemployment and productivity loss costs totaled nearly $13.5 billion for this population in MEPS in 2020.

Sensitivity analyses limiting the data sample to MEPS respondents with schizophrenia only did not meaningfully differ from those that included other psychotic disorders.

Relevant analysis results were inflated to 2024 $US as parameters used to estimate specific types of costs for community-dwelling individuals with schizophrenia in the overall cost model (eTable 11 and eTable 17).

Analysis Limitations

The per-person cost estimates derived from MEPS for community-dwelling individuals represent only the cost estimates for individuals with schizophrenia living in this setting. Per person cost estimates from MEPS may not be applicable to populations in other settings (e.g., long-term care, skilled nursing facilities, homeless, or incarcerated).

MEPS data was only available through 2015 for populations with schizophrenia spectrum disorders. Health care utilization and labor market changes since 2015 could impact overall cost estimates. The risk of this is mitigated by use of an excess cost estimate approach.

© 2026 Krasa HB et al. *JAMA Psychiatry*.

eAppendix 2. Model Parameters

Model parameters identified from literature reviews and empirical analyses described in section eAppendix 1 were used to estimate national and state costs of schizophrenia as outlined in the eFigure in eAppendix 1. Additional detail on identified parameters including parameter description, applicable model population, source reference values, adjusted model values, adjustment approach, and state-level adjustment, where applicable, are presented by model domain in sections S2.1 through S2.10.

S2.1 Model Input Parameters: Prevalence

National and setting-specific estimates of schizophrenia prevalence rates were abstracted from peer-reviewed literature for all settings. The percentage of adults with schizophrenia in independent households was estimated by subtracting the sum of those in the other settings (supportive housing, unhoused, long-term care or skilled nursing facility, and incarcerated). eTable 10 provides the original source values, adjustments to the source value, calculations used in the model, and relevant clarifications for calculations.

eTable 10: National and State Model Input Parameters: Prevalence

Population living situation or setting	National estimate parameters			State-level adjustment
	Parameter description	Source value	Model value	
General population	Total population, No.[15]	340,110,988	340,110,988	State total population[16]
General population, adults	Adult population, No.[15]	263,249,065	263,249,065	State adult population[16]
Schizophrenia population, adults	Adults with schizophrenia, %[17,18]	1.2% (age 18-65) adjusted to include >65	1.17%	No adjustment
	Adults with schizophrenia, No.	Calculated[a]	3,070,739	Calculated[a]
Community dwelling, independent household	Adult schizophrenia population in independent households, %	Calculated[b]	68.4%	Calculated[b]
	Adult schizophrenia population in independent households, No.	Calculated	2,100,079	Calculated
Community dwelling, supportive housing	Adult schizophrenia population in supportive housing, %[19]	18.6%	18.6%	No adjustment
	Adult schizophrenia population in supportive housing, No.	Calculated	571,157	Calculated
Long-term care and skilled nursing facilities	General population in LTC/SNF, No.[20]	1,400,000	1,400,000	Adjusted by state population percentage of national population
	General population in LTC/SNF, %	Calculated	0.53%	Calculated

© 2026 Krasa HB et al. *JAMA Psychiatry*.

Population living situation or setting	National estimate parameters			State-level adjustment
	Parameter description	Source value	Model value	
	LTC/SNF with schizophrenia, %[21]	11.0%	11.0%	No adjustment
	Adult schizophrenia population in LTC/SNF, %	Calculated	5.0%	Calculated
	Adult schizophrenia population in LTC/SNF, No.	Calculated	154,000	Calculated
Unhoused or Homeless	Adult population experiencing homelessness, No. [22]	541,484	541,484	State-specific data [22]
	Schizophrenia prevalence among homeless population, %[23]	18.8%	18.8%	Adjusted based on ratio of those within state who receive SSDI[24] to state homeless population relative to the national population
	Adult schizophrenia population experiencing homelessness, No.	Calculated[c]	101,799	Calculated[c]
	Adult schizophrenia population experiencing homelessness, %	Calculated	3.3%	Calculated
Incarcerated	Total correctional population, No. [25]	1,861,000[d]	1,861,000	State correctional population[25]
	Adult population incarcerated, %[25]	Calculated[e]	0.73%	Calculated
	Psychotic disorder prevalence among incarcerated, %[26]	Calculated[f]	7.7%	Calculated
	Adult schizophrenia population in prison or jail, No.	Calculated[g]	143,703	Calculated
	Adult schizophrenia population in prison or jail, %	Calculated[h]	4.68%	Calculated

Adult populations include age 18 and over. All percent source values noted as "Calculated" without a superscript were calculated as follows: [(parameter schizophrenia population/parameter general population)*100]. All number source values noted as "Calculated" without a superscript were calculated as follows: (percent of parameter population x overall parameter population number)
Abbreviations: LTC, Long-term care facility; SNF, Skilled nursing facility; SSDI, Social Security Disability Insurance
[a] Total adult population x % of total population with schizophrenia
[b] 1 – (the sum of the percent of those with schizophrenia who are homeless + LTC + incarcerated + structured residences)
[c] [percent of homeless population with schizophrenia x general homeless population number] / total US adult population
[d] Sum of state prisons + local jails + federal prisons and jails + territorial prisons + involuntary commitment
[e] Calculated with adult only population (overall incarcerated minus youth divided by adult population)
[f] Weighted average of 8.8% state and 3.2% federal
[g] Psychotic disorder prevalence among incarcerated, % x total correctional population
[h] Number of those incarcerated divided by total number with schizophrenia

© 2026 Krasa HB et al. *JAMA Psychiatry*.

S2.2 Model Input Parameters: Health Care

Direct health care costs were calculated for all settings, except for incarcerated. Health care costs for incarcerated individuals are included in the overall costs of incarceration. MEPS analysis estimates (see Section S1.3) are used for persons in a community setting (independent household or structured residence). Parameters abstracted during the targeted literature reviews inform excess health care costs for individuals experiencing homelessness and in long-term care or skilled nursing facilities. Schizophrenia-related health care costs across residential settings were calculated as the per-person differentials in costs between adults with schizophrenia and adults without the condition. eTable 11 provides the original source values, adjustments to the source value, calculations used in the model, and relevant clarifications for calculations.

eTable 11: National and State Model Input Parameters: Health Care Costs

Setting	Cost category	National estimate parameters				State-level adjustment
		Parameter description	Source value	Model value	Cost inflation scale/ adjustment	
Community dwelling, independent household & structured residence	Office-based visits	Office visit costs, excess per person[eTable 4]	$2,006	$2,230	PHCE	PW_PE
	Home health care	Home health care costs, excess per person[eTable 4]	$1,512	$1,681	PHCE	PW_PE
	Outpatient visits	Outpatient costs, excess per person[eTable 4]	$832	$925	PHCE	PW_PE
	Emergency room	Emergency room costs, excess per person[eTable 4]	$163	$181	PHCE	PW_PE
	Prescription drugs	Prescription drug costs, excess per person[eTable 4]	$4,560	$5,069	PHCE	PW_PE
	Out-of-pocket	Out-of-pocket costs, excess per person[eTable 4]	$315	$350	PHCE	PW_PE
	Inpatient hospital stay	Inpatient hospital costs, excess per person[eTable 4]	$2,162	$2,404	PHCE	PW_PE
	Overall health care	Total health care costs, excess per person	Calculated[a]	$12,840	PHCE	PW_PE
Unhoused or Homeless	Overall health care	Total health care costs, housed population[27]	$7,907	$8,527	PHCE	PW_PE
	Overall health care	Total health care costs, homeless population[27]	$19,623	$21,161	PHCE	PW_PE
	Overall health care	Schizophrenia homeless population, excess total health care cost ratio	Calculated[b]	1.48	n/a	Calculated[b]
	Overall health care	General population, average health care cost per person[28]	$6,765	$6,975	PHCE	PW_PE

© 2026 Krasa HB et al. *JAMA Psychiatry*.

Setting	Cost category	National estimate parameters				State-level adjustment
		Parameter description	Source value	Model value	Cost inflation scale/ adjustment	
	Overall health care	Schizophrenia population experiencing homelessness, excess health care cost per person	Calculated[c]	$10,334	n/a	Calculated[c]
Long-term care and skilled nursing facilities	LTC/SNF health care	Schizophrenia population, excess overall health care costs in LTC/SNF[29]	$1,496,000,000	n/a	n/a	n/a
	LTC/SNF health care	Schizophrenia population in LTC/SNF, No.[29]	195,892	n/a	n/a	n/a
	LTC/SNF health care	Schizophrenia population, per person excess health care costs in LTC/SNF	$7,637[d]	$8714	PHCE	PW_PE

$US in model value column are adjusted to $2024
Abbreviations: n/a, not applicable; PHCE, Personal Health Care Expenditure Index; PW_PE, Center for Medicare and Medicaid Services physician wage (PW) and practice expense (PE) Geographic Practice Cost Indices; LTC, Long-term care facility; SNF, Skilled nursing facility.
[a] [(Schizophrenia population in independent households + Schizophrenia population in supportive housing)/(MEPS estimated total health care cost differential)]
[b] [Total health care cost homeless population minus total health care cost general population]/ total health care cost general population
[c] [Schizophrenia homeless population excess total health care cost ratio] x [general population (community-dwelling) total health care costs]
[d] [Schizophrenia population excess overall health care costs in LTC/SNF] / [estimated number with schizophrenia in LTC/SNF]

© 2026 Krasa HB et al. *JAMA Psychiatry*.

S2.3 Model Input Parameters: Social Security Disability Benefits

Enrollment in two types of social security disability benefits, social security disability income (SSDI) and supplemental security income (SSI), offered by the Social Security Administration was gathered at the national and state level, including the District of Columbia. Schizophrenia-related disability benefit costs across residential settings were calculated by applying general-population month benefit to the additional number of adults with schizophrenia receiving benefits. eTable 12 provides the original source values, adjustments to the source value, calculations used in the model, and relevant clarifications for calculations.

eTable 12: National and State Model Input Parameters: Social Security Disability Benefits

Setting	Cost category	National estimate parameters				State-level adjustment
		Parameter description	Source value	Model value	Cost inflation scale/ adjustment	
All settings	SSDI	General population receiving SSDI, No.[24]	10,082,479	10,082,479	n/a	State-specific data[24]
		Schizophrenia population receiving SSDI, No.[24]	421,602	421,602	n/a	State-specific data [24]
		Average monthly per person SSDI, schizophrenia population[24]	$1,095	$1,173	CPI_W	No adjustment
		Schizophrenia population receiving SSDI, %	Calculated[a]	13.73%	n/a	Calculated[a]
		General population receiving SSDI, %	Calculated[a]	3.83%	n/a	Calculated[a]
		Excess receiving SSI due to schizophrenia	Calculated[b]	303,992	n/a	Calculated[b]
All settings	SSI	General population receiving SSI, No.[24]	3,186,435	3,186,435	n/a	State-specific data[24]
		Schizophrenia population receiving SSI, No.[24]	268,285	268,285	n/a	State-specific data[24]
		Average monthly per person SSI, schizophrenia population[24]	$285	$306	CPI_W	No adjustment
		Schizophrenia population receiving SSI, %	Calculated[a]	8.74%	n/a	Calculated[a]
		General population receiving SSI, %	Calculated[a]	1.21%	n/a	Calculated[a]
		Excess receiving SSI due to schizophrenia	Calculated[a]	231,116	n/a	Calculated[a]

$US in model value column are adjusted to $2024

© 2026 Krasa HB et al. *JAMA Psychiatry*.

Abbreviations: n/a, not applicable; SSDI, social security disability insurance; SSI, supplemental security income; CPI_W, Consumer Price Index – Urban Wage Earners and Clerical Workers.
[a] (number receiving benefit/number in the population)*100 [b] [(General population with benefits – schizophrenia population with benefits)/ US population with schizophrenia]

© 2026 Krasa HB et al. *JAMA Psychiatry*.

S2.4 Model Input Parameters: Supportive Housing and Homelessness Remediation

Literature-based estimations informed the costs of long-term care/skilled-nursing facilities and the costs of homelessness remediation. To avoid over-estimation, cost offsets based upon the individual US poverty threshold were applied to final estimates. Schizophrenia-related supportive housing and homelessness remediation costs were calculated by applying general-population costs to the additional number of adults with schizophrenia incurring costs the setting. eTable 13 provides the original source values, adjustments to the source value, calculations used in the model, and relevant clarifications for calculations.

eTable 13: National and State Model Input Parameters: Supportive Housing and Homelessness

Setting	Cost category	National estimate parameters				State-level adjustment
		Parameter description	Source value	Model value	Cost inflation scale/ adjustment	
Community-dwelling, structured residence	Supportive housing	Per-person cost of adult residential facility, annual[30]	$25,806	$32,726	PCE	PW_PE
	Housing cost-offset	US poverty threshold, single person[31]	$15,060	$15,060	No adjustment	No adjustment
Long-term care/skilled nursing facility	Supportive housing	Per-person monthly cost of semi-private room in long-term care facility[32]	$8,669	$8,938	PHCE	PW_PE
	Housing cost-offset	US poverty threshold, single person[31]	$15,060	$15,060	No adjustment	No adjustment
Unhoused or homeless	Homeless-ness remediation	Homeless population, annual cost of shelter stays[30]	$14,064	$17,835	PCE	PW_PE
	Housing cost-offset	US poverty threshold, single person[31]	$15,060	$15,060	No adjustment	No adjustment

$US in model value column are adjusted to $2024

Abbreviations: n/a, not applicable; PCE, Personal Consumption Expenditures Price Index; PHCE, Personal Health Care Expenditure Index; PW_PE, Center for Medicare and Medicaid Services physician wage (PW) and practice expense (PE) Geographic Practice Cost Indices

© 2026 Krasa HB et al. *JAMA Psychiatry*.

S2.5 Model Input Parameters: Justice System Interactions

In addition to costs due to incarceration, the model also accounted for judicial system contact. Schizophrenia-related justice system interaction costs were calculated as the per-person differentials in costs between adults with schizophrenia and adults without the condition with by type of system interaction. eTable 14 provides the original source values, adjustments to the source value, calculations used in the model, and relevant clarifications for calculations.

eTable 14: National and State Model Input Parameters: Justice System Interactions

Setting	Cost category	National estimate parameters				State-level adjustment
		Parameter description	Source value	Model value	Cost inflation scale/ adjustment	
Community- dwelling, independent household and structured residence; unhoused or homeless	Justice system interactions	Schizophrenia population annual contact with justice system, %[33]	46.0%	46.0%	n/a	No adjustment
	Justice system interactions	General population annual contact with justice system, %	Calculated[a]	7.2%	n/a	Calculated[a]
	Justice system interactions	Schizophrenia population annual per person cost of justice system interactions[33]	$1,429	$2,342	PCE	PW_PE
Incarcerated	Incarceration	Annual cost of incarcerating mentally ill inmate[34]	$75,000[b]	$75,543	PCE	PW_PE
	Incarceration cost-offset	US poverty threshold, single person[31]	$15,060	$15,060	No adjustment	No adjustment

$US in model value column are adjusted to $2024
Abbreviations: PCE, Personal Consumption Expenditures Price Index; PW_PE, Center for Medicare and Medicaid Services physician wage (PW) and practice expense (PE) Geographic Practice Cost Indices.
[a] (% of schizophrenia population with justice system contact) / (% of people with schizophrenia incarcerated / % of people incarcerated)
[b] The population weighted average of Texas, Florida, and Washington

© 2026 Krasa HB et al. *JAMA Psychiatry*.

S2.6 Model Input Parameters: Reduced Quality of Life

The impact of a reduced quality of life (i.e., morbidity) estimated to have been incurred by those with schizophrenia is converted to $US using a willingness to pay approach where the value of a statistical year of life is set at $100,000 in the base case. Schizophrenia related costs across residential settings were calculated as the per-person differentials in quality adjusted life years (QALY) between adults with schizophrenia and adults without the condition times the value of a QALY for total number with schizophrenia less mortality. eTable 15 provides the original source values, adjustments to the source value, calculations used in the model, and relevant clarifications for calculations.

eTable 15: National and State Model Input Parameters: Reduced Quality of Life

Setting	Cost category	National estimate parameters				State-level adjustment
		Parameter description	Source value	Model value	Cost inflation scale/ adjustment	
All settings	Quality of life	Value of a QALY[35]	$100,000	$100,000	No adjustment	No adjustment
	Quality of life	General adult population, QALY[36]	0.868	0.868	n/a	No adjustment
	Quality of life	Schizophrenia population, QALY[37]	0.730	0.730	n/a	No adjustment

Abbreviations: n/a, not applicable; QALY, Quality adjusted life year.

© 2026 Krasa HB et al. *JAMA Psychiatry*.

S2.7 Model Input Parameters: Shortened Life Expectancy

The indirect cost of increased mortality due to the elevated risk of death in schizophrenia was calculated as the present value of all future quality adjusted lost years of life among those estimated to die due to the disease in 2024. Schizophrenia related costs across residential settings were calculated as the differential in mortality rate between adults with schizophrenia and adults without the condition times the discounted value of reduced life expectancy. eTable 16 provides the original source values, adjustments to the source value, calculations used in the model, and relevant clarifications for calculations.

eTable 16: National and State Model Input Parameters: Shortened Life Expectancy

Setting	Cost category	National estimate parameters				State estimate parameter adjustment
		Parameter description	Source value	Model value	Cost inflation scale/ adjustment	
All settings	Life expectancy	Value of a QALY[35]	$100,000	$100,000	No adjustment	No adjustment
	Life expectancy	General adult population, QALY[36]	0.868	0.868	n/a	No adjustment
	Life expectancy	Schizophrenia population, QALY[36]	0.730	0.730	n/a	No adjustment
	Life expectancy	General population, annual overall death rate, %[38]	0.959%	0.959%	n/a	State specific death rate[38]
	Life expectancy	Standardized mortality ratio for schizophrenia vs. general population[39]	2.6	2.6	n/a	No adjustment
	Life expectancy	Schizophrenia population, annual overall death rate	Calculated	2.49%	Calculated	Calculated
	Life expectancy	Years of potential life lost per schizophrenia death[40]	14.50	14.50	n/a	No adjustment
	Life expectancy	Discount rate	3%	3%	n/a	No adjustment

Abbreviation: QALY, Quality adjusted life year.

© 2026 Krasa HB et al. *JAMA Psychiatry*.

S2.8 Model Input Parameters: Non-employment and Reduced Wages

Lost wages due to underemployment and unemployment were used to estimate indirect costs due to schizophrenia. Schizophrenia related costs across residential settings were calculated as the per-person differentials between adults with schizophrenia and adults without the condition for non-employment and reduced wages. eTable 17 provides the original source values, adjustments to the source value, calculations used in the model, and relevant clarifications for calculations.

eTable 17: National and State Model Input Parameters: Non-employment and Reduced Wages

Setting	Cost category	National estimate parameters				State estimate parameter adjustment
		Parameter description	Source value	Model value	Cost inflation scale/ adjustment	
All settings	Non-employment	General population employment rate,%[eTable 2]	63.6%	63.6%	n/a	State's employment rate relative to the national rate
	Non-employment	Schizophrenia population excess lost income from non-employment[eTable 5]	$39,017	$45,295	HCOMP-BS	Average wage of state relative to the national average
	Reduced wages	Schizophrenia population excess lost income from reduced wages[eTable 5]	$12,727	$14,775	HCOMP-BS	Average wage of state relative to the national average
Community dwelling, independent households and structured residences	Non-employment	Schizophrenia population employment rate in independent households, %[eTable 2]	37.3%	37.3%	n/a	State's employment rate relative to the national rate
	Non-employment	Schizophrenia population excess non-employed in independent households, No.	Calculated[a]	553,161	n/a	Calculated[a]
	Reduced wages	Schizophrenia population employed in independent households, No.	Calculated[b]	782,700	n/a	Calculated[b]
	Non-employment	Schizophrenia population employment rate in structured residences, %[41, eTable 2]	12.7%	12.7%	n/a	State's employment rate relative to the national rate
	Non-employment	Schizophrenia population excess non-employed in structured residences, No.	Calculated[c]	290,776	n/a	Calculated[c]
	Reduced wages	Schizophrenia population employed in independent households, No.	Calculated[b]	72,537	n/a	Calculated[b]

© 2026 Krasa HB et al. *JAMA Psychiatry*.

Setting	Cost category	National estimate parameters				State estimate parameter adjustment
		Parameter description	Source value	Model value	Cost inflation scale/ adjustment	
Unhoused or homeless, incarcerated	Non-employment	Schizophrenia population excess non-employed, No.	Calculated[a]	100,123	n/a	Calculated[a]

$US in model value column are adjusted to $2024
Abbreviations: HCOMP-BS, Business Sector: Hourly Compensation for All Workers
a Number unemployed with schizophrenia in setting – number unemployed without schizophrenia in setting
b [Number with schizophrenia in setting] x [schizophrenia population employment rate in setting]
c [(1 - schizophrenia population employment rate in setting) x (number with schizophrenia in setting)] – [(1 – general population employment rate) x (number with schizophrenia in setting)]

© 2026 Krasa HB et al. *JAMA Psychiatry*.

S2.9 Model Input Parameters: Caregiver Burden

Economic burden associated with informal caregivers for individuals with schizophrenia (i.e. caregiver burden) included four cost categories: unpaid wages (i.e. uncompensated labor for time providing care), lost productivity (i.e., absenteeism or presenteeism), caregiver health care, and out-of-pocket costs for everyday expenses and significant life events. Uncompensated labor was calculated by multiplying caregiver hours spent by the mean US wage applied to the number of caregivers incurring costs in the setting. Productivity loss and health care costs were calculated as the per-person differentials between adults with schizophrenia and adults without the condition. Caregiver out-of-pocket costs were calculated by applying costs to the population of caregivers in that setting. eTable 18 provides the original source values, adjustments to the source value, calculations used in the model, and relevant clarifications for calculations.

eTable 18: National and State Model Input Parameters: Caregiver Unpaid Wages and Other Impacts

Setting	Cost category	National estimate parameters				State estimate parameter adjustment
		Parameter description	Source value	Model value	Cost inflation scale/ adjustment	
Community dwelling, independent households and structured residences	Caregiver burden	Schizophrenia population eligible for a caregiver, No.	Calculated[a]	2,671,237	n/a	Calculated[a]
	Caregiver burden	Schizophrenia population in the community with a caregiver, %[42]	65.0%	65.0%	n/a	n/a
	Caregiver burden	Primary caregivers for schizophrenia population, No.	Calculated[b]	1,736,304	n/a	Calculated[b]
	Caregiver unpaid wages	Average hours per week per caregiver providing direct care[43]	36.1	36.1	n/a	n/a
	Caregiver unpaid wages	Average hourly US wage[44]	$32.09	$32.09	No adjustment	Average wage of state relative to the national average[44]
	Caregiver health care	Schizophrenia caregiver, excess annual direct health care cost[45]	$5,993	$7,822	PHCE	PW_PE
	Caregiver productivity loss	Excess annual work absenteeism cost per caregiver[45]	$1,282	$1,934	HCOMP-BS	Average wage of state relative to the national average
	Caregiver productivity loss	Excess annual work presenteeism cost per caregiver[45]	$ 2,532	$3,819	HCOMP-BS	Average wage of state relative to the national average

© 2026 Krasa HB et al. *JAMA Psychiatry*.

Setting	Cost category	National estimate parameters				State estimate parameter adjustment
		Parameter description	Source value	Model value	Cost inflation scale/ adjustment	
	Caregiver OOP costs	Monthly OOP paid by caregivers for adult care recipient everyday expenses[43]	$1,575[c,d]	$1,661	PHCE	No adjustment
	Caregiver OOP costs	Annual OOP paid by caregivers for adult care recipient significant life event expenses[43]	$1,268[e,f]	$1,303	PHCE	No adjustment
Community dwelling, independent households	Caregiver OOP cost offsets	Monthly OOP expenses paid by caregiver considered transfer costs	$1,015[g]	$1,071	PHCE	No adjustment

$US in model value column are adjusted to $2024. Individuals with schizophrenia residing in long-term care or skilled nursing facilities, incarcerated, or homeless were assumed not to have a caregiver for the purposes of this model.

Abbreviations: n/a, not applicable; PHCE, Personal Health Care Expenditure Index; PW_PE, Center for Medicare and Medicaid Services physician wage (PW) and practice expense (PE) Geographic Practice Cost Indices; HCOMP-BS, Hourly Compensation for All Workers OOP, Out-of-pocket

[a] [Number with schizophrenia] – [Number with schizophrenia in long-term care/skilled nursing facilities, incarcerated, and homeless]

[b] [Number with schizophrenia eligible for a caregiver] x [schizophrenia population percent with a caregiver]

[c] Monthly everyday expenses included payments for cost-of-living expenses such as food, transportation, clothing, pocket money, personal items, cigarettes, other substances, and housing and well as other expenses potentially related to schizophrenia such as medication, mental health treatment, other medical expenses, and property damage.

[d] [Average monthly OOP for everyday expenses ($1810) x number of caregivers with expenses (n=174)] / [total number of caregivers surveyed (n=200)]

[e] Annual expenses for significant life events not paid for by insurance or other program included costs relating to substance use treatment, suicide attempts, arrest, incarceration, other legal services, education and employment support services, and homelessness. OOP expenses for psychiatric hospitalization in this study were excluded as they are assumed to be included in the MEPS analysis health care expense OOP costs.

f [Average annual OOP for MLE ($3990) x number with expenses (n=110)] / [total number surveyed (n=200)] minus [OOP for psychiatric hospitalization ($2,233) x number with expense (n=83)]/ [total number surveyed (n=200)]

[g] Sum of costs of food, transportation, clothing, pocket money, personal items, cigarettes, other substances, and housing of mean cost per category times the percent of sample that reported the cost of living expenses: (129/200 reported average food cost of $341)+(118/200 reported average transportation cost of $265) +(87/200 reported average clothing cost of $332) +(86/200 reported average pocket money cost of $327) +(78/200 reported average personal items cost of $250) +(55/200 reported average housing cost of $670) +(42/200 reported average cigarette cost of $81) +(39/200 reported average other substances cost of $296)

S2.10 Model Input Parameters: Lifetime Cost of Schizophrenia Scenario

The discussion section presents a "what-if" scenario that frames the results of this cross-sectional analysis in an alternative way. If mortality burden (i.e., the estimated costs of lost years of life) was removed and the remaining burden divided by the total number of adults with schizophrenia, this per person value could be used as an annual average burden over a lifetime. From this the total and present value of lifetime costs could be computed. eTable 19 provides the original source values, adjustments to the source value, calculations used in the model, and relevant clarifications for calculations.

© 2026 Krasa HB et al. *JAMA Psychiatry*.

eTable 19: National and State Model Input Parameters: Lifetime Cost of Schizophrenia Scenario

Setting	Cost category	National estimate parameters				State estimate parameter adjustment
		Parameter description	Source value	Model value	Cost inflation scale/ adjustment	
All settings	Lifetime costs	Age of adult with schizophrenia	18	18	n/a	No adjustment
		General population life expectancy at age 18[46]	59.1	59.1	n/a	No adjustment
		Life expectancy, schizophrenia population at age 18	44.6	44.6	n/a	No adjustment
		Average excess costs per person, per year (Excluding Morbidity and Mortality)	$103,980	$103,980	No adjustment	No adjustment
		Discount rate[eTable 16]	3%	3%	n/a	No adjustment
		Lifetime estimated costs	$4,496,034	n/a	No adjustment	No adjustment
		Present value of lifetime costs	$2,461,112	n/a	No adjustment	No adjustment

© 2026 Krasa HB et al. *JAMA Psychiatry*.

eReferences.

1. U.S. Bureau of Economic Analysis. Personal consumption expenditures: Services: Health care [DHLCRC1Q027SBEA]. Federal Reserve Bank of St. Louis; 2024. https://fred.stlouisfed.org/series/DHLCRC1Q027SBEA. Accessed 23 July 2024.
2. Organization for Economic Co-operation and Development. Consumer Price Index for All Urban Wage Earners and Clerical Workers: All Items in U.S. City Average [CWUR0000SA0]. Federal Reserve Bank of St. Louis; 2024. https://fred.stlouisfed.org/series/CWUR0000SA0. Accessed 23 July 2024.
3. U.S. Bureau of Labor Statistics. Business Sector: Hourly Compensation for All Workers [HCOMPBS]. Federal Reserve Bank of St. Louis,; 2024. https://fred.stlouisfed.org/series/HCOMPBS. Accessed 23 July 2024.
4. U.S. Bureau of Economic Analysis. Personal consumption expenditures: Services: Health care (chain-type price index) [DHLCRG3Q086SBEA]. Federal Reserve Bank of St. Louis; 2024. https://fred.stlouisfed.org/series/DHLCRG3Q086SBEA. Accessed 23 July 2024.
5. Centers for Medicare & Medicaid Services. *Geographic Practice Cost Indices (GPCIs)*. 2023. 6 November 2023. Accessed 5 November 2024.
6. U.S. Bureau of Labor Statistics. May 2023 State Occupational Employment and Wage Estimates.. https://www.bls.gov/oes/2023/may/oessrcst.htm. Accessed 13 September 2024
7. Agency for Healthcare Research and Quality. Medical Expenditure Survey Household Component. Department of Health and Human Services. 2020. https://meps.ahrq.gov/mepsweb/survey_comp/household.jsp. Accessed October 20, 2024.
8. Healthcare Cost & Utilization Project. Clinical Classifications Software (CCS) for ICD-9-CM. Agency for Healthcare Research and Quality (AHRQ). https://hcup-us.ahrq.gov/toolssoftware/ccs/ccs.jsp. Accessed October 3, 2024.
9. Agency for Healthcare Research and Quality. MEPS HC-180: 2015 Medical Conditions (Section 2.5.2.8 Clinical Classification Codes). Department of Health and Human Services. 2020. https://meps.ahrq.gov/data_stats/download_data/pufs/h180/h180doc.shtml#Medical2.5.2. Accessed October 3, 2024.
10. Agency for Healthcare Research and Quality (AHRQ). Appendix A - Clinical Classification Software-DIAGNOSES (January 1980 through September 2015). 2016. 24 March 2016. https://hcup-us.ahrq.gov/toolssoftware/ccs/AppendixASingleDX.txt. Accessed October 3, 2024.
11. Desai PR, Lawson KA, Barner JC, Rascati KL. Estimating the direct and indirect costs for community-dwelling patients with schizophrenia. *Journal of Pharmaceutical Health Services Research*. 2013;4(4):187-194. doi:10.1111/jphs.12027
12. Iacus SM, King G, Porro G. Causal Inference without Balance Checking: Coarsened Exact Matching. *Political Analysis*. 2012;20(1):1-24. doi:10.1093/pan/mpr013
13. U.S. Bureau of Labor Statistics. Employer costs for Employee Compensation – March 2019. Released June 18, 2019. https://www.bls.gov/news.release/archives/ecec_06182019.pdf. Accessed September 5, 2020.
14. U.S. Bureau of Labor Statistics. Consumer Price Index (CPI). 2020. https://www.bls.gov/cpi/. Accessed September 5, 2020.
15. U.S. Census Bureau. Population Estimates, July 1, 2024 (V2024). Quick Facts https://www.census.gov/quickfacts/fact/table/US#. Accessed January 15, 2025.
16. U.S. Census Bureau. ACS Demographics and Housing Estimates. *American Community Survey, ACS 1-Year Estimates Data Profiles, Table DP05*. 2022. https://data.census.gov/table/ACSDP5Y2023.DP05?q=DP05:+ACS+Demographic+and+Housing+Estimates&g=010XX00US$8600000. Accessed January 15, 2025.
17. Ringeisen H, Edlund M, Guyer H, et al. Prevalence of Past-Year Mental and Substance Use Disorders, 2021-2022. *Psychiatr Serv*. Aug 1 2025;76(8):720-728. doi:10.1176/appi.ps.20240329

18. U.S. Census Bureau. National Demographic Analysis Tables: 2020. https://www.census.gov/data/tables/2020/demo/popest/2020-demographic-analysis-tables.html. . Accessed January 15, 2025.

19. Swanson JW, Swartz MS, Dorn RAV, et al. A National Study of Violent Behavior in Persons With Schizophrenia. *Arch Gen Psychiatry*. 2006;63:490-499. doi:10.1001/archpsyc.63.5.490

20. Hado E, Komisar H. Fact Sheet: Long-Term Services and Supports. AARP Public Policy Institute; 2019. August 2019. doi:10.26419/ppi.00079.001 Accessed May 14, 2024.

21. Fashaw S, Chisholm L, Mor V, et al. Inappropriate Antipsychotic Use: The Impact of Nursing Home Socioeconomic and Racial Composition. *J Am Geriatr Soc*. 2020;68(3):630-636. doi:10.1111/jgs.16316

22. Sousa Td, Andrichik A, Prestera E, Rush K, Tano C, Wheeler M. *The 2023 Annual Homelessness Assessment Report (AHAR) to Congress*. 2023. https://www.huduser.gov/portal/publications/2023-ahar-part-1-pit-estimates-of-homelessness.html. Accessed October 14, 2024.

23. Ayano G, Tesfaw G, Shumet S. The prevalence of schizophrenia and other psychotic disorders among homeless people: a systematic review and meta-analysis. *BMC Psychiatry*. 2019. doi:10.1186/s12888-019-2361-7

24. Social Security Administration. Annual Statistical Report on the Social Security Disability Insurance Program, 2023. https://www.ssa.gov/policy/docs/statcomps/di_asr/2023/di_asr23.pdf. Accessed November 5, 2024.

25. Sawyer W, Wagner P. Mass Incarceration: The Whole Pie 2024. Prison Policy Initiative; 2024. March 14, 2024. https://www.prisonpolicy.org/reports/pie2024.html. Accessed September 3, 2024.

26. Maruschak LM, Bronson J, Alper M. Survey of Prison Inmates, 2016 - Indicators of Mental Health Problems Reported by Prisoners. 2021.

27. Koh KA, Racine M, Gaeta JM, et al. Health Care Spending And Use Among People Experiencing Unstable Housing In The Era Of Accountable Care Organizations. *Health Aff (Millwood)*. 2020;39(2):214-223. doi:10.1377/hlthaff.2019.00687

28. Hernandez-Viver A, Mitchell EM. Concentration of Healthcare Expenditures and Selected Characteristics of People with High Expenses, United States Civilian Noninstitutionalized Population, 2018-2022. *Statistical Brief (Medical Expenditure Panel Survey (US))*. Department of Health and Human Services; 2025 Mar. https://www.ncbi.nlm.nih.gov/books/NBK613741/. Accessed April 1, 2025

29. Kadakia A, Catillon M, Fan Q, et al. The Economic Burden of Schizophrenia in the United States. *Journal of Clinical Psychiatry*. 2022;83. doi:10.4088/JCP.22m14458

30. Culhane DP, An S. Estimated Revenue of the Nonprofit Homeless Shelter Industry in the United States: Implications for a More Comprehensive Approach to Unmet Shelter Demand. *Housing Policy Debate*. 2022;32:823-836. doi:10.1080/10511482.2021.1905024

31. Department of Health and Human Services. Annual Update of the HHS Poverty Guidelines. 2024;89(11).

32. Genworth. Cost of Care Survey 2023. https://www.genworth.com/aging-and-you/finances/cost-of-care. Accessed May 1, 2024.

33. Ascher-Svanum H, Nyhuis AW, Faries DE, Ball DE, Kinon BJ. Involvement in the US criminal justice system and cost implications for persons treated for schizophrenia. *BMC Psychiatry*. 2010. doi:10.1186/1471-244X-10-11

34. Torrey E, Zdanowicz M, Kennard A, et al. The Treatment of Persons with Mental Illness in Prisons and Jails: A State Survey. 2014. https://tac2.nonprofitsoapbox.com/storage/documents/treatment-behind-bars/treatment-behind-bars.pdf. Accessed April 24, 2024.

35. Institute for Clinical And Economic Review. Guide to Understanding Health Technology Assessment (HTA). 2018.

36. Sullivan PW, Ghushchyan V. Preference-Based EQ-5D Index Scores for Chronic Conditions in the United States. *Med Decis Making*. 2006;26(4):410-420. doi:10.1177/0272989X06290495

37. Aceituno D, Pennington M, Iruretagoyena B, Prina AM, McCrone P. Health State Utility Values in Schizophrenia: A Systematic Review and Meta-Analysis. *Value Health*. 2020;23(9):1256-1267. doi: 10.1136/ebmental-2019-300089

38. Centers for Disease Control and Prevention. National Vital Statistics System, Mortality 2018-2022. http://wonder.cdc.gov/ucd-icd10-expanded.html. Accessed May 9, 2024.

39. Saha S, Chant D, McGrath J. A Systematic Review of Mortality in Schizophrenia: Is the Differential Mortality Gap Worsening Over Time? *Arch Gen Psychiatry*. 2007;64(10):1123-1131. doi:10.1001/archpsyc.64.10.1123

40. Hjorthøj C, Stürup AE, McGrath JJ, Nordentoft M. Years of potential life lost and life expectancy in schizophrenia: a systematic review and meta-analysis. *Lancet Psychiatry*. 2017;4:295-301. doi:10.1016/S2215-0366(17)30078-0

41. Strassnig M, Kotov R, Fochtmann L, Kalin M, Bromet EJ, Harvey PD. Associations of independent living and labor force participation with impairment indicators in schizophrenia and bipolar disorder at 20-year follow-up. *Schizophrenia Research*. 2018. doi:10.1016/j.schres.2018.02.009

42. Cloutier M, Aigbogun MS, Guerin A, et al. The Economic Burden of Schizophrenia in the United States in 2013. *J Clin Psychiatry*. 2016;77(6):764-771. doi: 10.1136/ebmental-2019-300089

43. Krasa H, Birch K, Eskew F, Frangiosa T, Palsgrove A, Maravic MC. Quantifying the societal impacts of schizophrenia: A survey of caregivers. *Psychiatric Research and Clinical Practice*; 2025. Article in Press. doi:10.1176/rcp2.70028

44. U.S. Bureau of Labor Statistics. May 2023 National Occupational Employment and Wage Estimates. https://www.bls.gov/oes/current//oes_nat.htm. Accessed October 3, 2024.

45. Csoboth C, Witt EA, Villa KF, O'Gorman C. The humanistic and economic burden of providing care for a patient with schizophrenia. *International Journal of Social Psychiatry*. 2015;61(8):754-761. doi:10.1177/0020764015577844

46. Arias E, Xu J, Kochanek K. United States Life Tables, 2021. *Natl Vital Stat Rep*. 2023;72(12):1-64.
