# Supplementary material for: National and State Societal Costs of Schizophrenia in the US in 2024
Source: JAMA Psychiatry. 2026 Jan 28;83(4):341–52. doi: 10.1001/jamapsychiatry.2025.4383 (PMC12853289; doi:10.1001/jamapsychiatry.2025.4383)
Supplement: Supplement 2. — Data Sharing Statement. [file jamapsychiatry-e254383-s002.pdf]

Data Sharing Statement

Krasa. National and State Societal Costs of Schizophrenia in the US in 2024. *JAMA Psychiatry*. Published January 28, 2026. doi:10.1001/jamapsychiatry.2025.4383

Data
Data available: Yes
Data types: Data dictionary
How to access data: Available in online only supplement of the article.
When available: With publication

Supporting Documents
Document types: None

Additional Information
Who can access the data: Researchers whose proposed use of the data has been approved.
Types of analyses: For specified purpose. Alternative data inputs into the existing model framework to generate ..... Model used in to estimate the societal costs of schizophrenia will be available as an interactive interface and accessible through https://sczaction.org/ following publication.
Mechanisms of data availability: Use of the societal costs model will be freely available. Alterations or supportive research requiring access to the model framework will be made available after approval of a proposal.
